# malariasimple: An R package for fast simulations of malaria transmission

**DOI:** 10.1371/journal.pcbi.1013687

**Published:** 2026-09-01

**Authors:** Debbie Shackleton, Neil Ferguson, Lucy Okell, Tom Churcher, Pete Winskill

**Affiliations:** MRC Centre for Global Infectious Disease Analysis, School of Public Health, Imperial College London, London, United Kingdom; University of Melbourne, AUSTRALIA

## Abstract

Process-based malaria transmission models are important tools for evaluating intervention strategies, quantifying uncertainty, and informing malaria control policy. Individual-based models such as *malariasimulation* are computationally demanding, which limits their practicality for applications that require large numbers of simulation runs. In this paper we present *malariasimple*, a simplified, compartmental model implemented as an R package which approximates the epidemiological structure and parameter definitions of *malariasimulation* while operating at a fraction of the computational cost. Across a range of transmission intensities and intervention scenarios, *malariasimple* closely reproduces key outputs of *malariasimulation* while reducing runtimes by up to 99.6%. Its computational efficiency enables full Bayesian parameter inference, allowing estimation of complete posterior distributions. *malariasimple* provides a fast, flexible, and mechanistically consistent addition to the Imperial College London Malaria Model framework, bridging the gap between computational efficiency and epidemiological realism. The *malariasimple* R package is freely available for download at https://github.com/mrc-ide/malariasimple.

## Author summary

Malaria remains a major global health challenge, and choosing how best to use limited resources for control is difficult. Mathematical models can help by testing possible intervention strategies before they are used in the real world. However, detailed malaria models that track individuals are computationally slow, making it impractical to run the thousands of simulations needed to rigorously quantify uncertainty in model predictions. This is a significant limitation, because data on malaria transmission are often sparse or imprecise, and understanding the range of plausible outcomes is essential for sound decision-making. We developed *malariasimple*, a new R package that provides a faster, simplified version of *malariasimulation*, an established individual-based malaria transmission model. Our model retains the essential biology of how malaria spreads between humans and mosquitoes, as well as the effects of widely used control interventions such as insecticide-treated bed nets and preventive drug treatments. We show that *malariasimple* produces outputs that are very similar to the more detailed model while reducing runtimes by up to 99.6%. This speed makes it practical to fit the model to data using Bayesian methods and to quantify uncertainty in predictions. *malariasimple* therefore offers a useful tool for researchers and policy analysts who need fast, interpretable malaria simulations.

## Introduction

Progress in reducing malaria transmission has stalled while the global malaria funding gap has widened [1], making it essential to maximise the impact of every dollar invested. Malaria control relies on a portfolio of prevention measures and prompt diagnosis with effective treatment, but deciding how best to deploy and combine these tools under budget constraints is challenging. Process-based transmission models help to address this need: relative to field studies, they provide fast, low-cost evaluations of intervention portfolios, allow exploration of strategies in future contexts such as climate change, and enable counterfactuals that would be impractical, unethical, or too slow to test empirically. In data-poor settings, mechanistic models supply the structure needed for prediction and generalisation.

The Imperial College Malaria Model framework [mrc-ide.github.io/malariaverse] is a suite of interconnected malaria transmission models developed to address diverse research and policy questions across epidemiological settings. At its core is *malariasimulation* [2], an open-source, individual-based model capable of representing a wide range of transmission contexts and intervention scenarios. Its individual-based structure allows detailed representation of heterogeneity and non-linear interactions that underpin malaria dynamics, making it particularly powerful for exploring complex intervention strategies and elimination pathways. However, this level of detail also comes at a high computational cost.

Key model parameters are often unknown in modelling scenarios and must be fitted to available data. In many settings, data may be sparse, unevenly distributed, and variable in quality, particularly across sub-Saharan Africa where malaria burden is highest [1]. Hence, in many scenarios, there will be substantial uncertainty in the true value of these parameters. Explicitly quantifying and propagating this uncertainty, as well as incorporating prior information is important to ensure model outputs reflect both the strength and limitations of the available evidence. However, common approaches which permit this, such as Bayesian inference, typically involve a large number of simulation runs which is prohibitive for computationally expensive individual-based models.

There is a need for an approximation of *malariasimulation* that can achieve substantially shorter simulation runtimes. A faster model would enable the use of Bayesian inference methods to estimate full posterior distributions of unknown parameters, explore parameter correlations, and propagate both process and observation uncertainty through to model predictions. One approach that has gained popularity in recent years is the use of emulators to create fast approximations of individual-based transmission models (e.g., [3–7]). These emulators are generally trained on many runs of the original model across the input parameter space and, once trained, can generate outputs almost instantaneously, enabling rapid Bayesian calibration and uncertainty analysis. However, this approach often comes at the cost of a computationally intensive upfront training stage and is fundamentally black-box in nature. This can make it less straightforward for users to explore modified model structures, for example by introducing a novel intervention or incorporating sensitivity to a particular environmental variable, as the emulator may need to be retrained.

In this paper, we present *malariasimple*, a simplified, discrete-time compartmental model implemented as an R package and designed as an approximation of *malariasimulation*. The model aims to preserve the core epidemiological structure, parameter definitions, and mechanistic logic of the full individual-based model while remaining computationally light enough to perform inference directly. Its mechanistic nature makes it easier to interpret, modify, and extend for users who wish to explore both parameter uncertainty and alternative model formulations. Further, by maintaining full compatibility with *malariasimulation*, *malariasimple* enables users to move seamlessly between fast exploratory analyses and detailed individual-based simulations within a consistent modelling framework.

The objective of this paper is to introduce *malariasimple* as a new open-source addition to the Imperial College Malaria Model framework, outlining its key functions, structure, and limitations. We also demonstrate its practical application through an example use case in which the model is fitted to prevalence data from a synthetic scenario using MCMC methods to produce posterior distributions for unknown parameters.

## Design and implementation

*malariasimple* is a discrete-time compartmental model of malaria transmission that captures the coupled dynamics of human and mosquito populations. The model is formulated using difference equations with state variables updated at fixed time steps, and converges to the equivalent differential equation formulation as the time step length approaches zero. *malariasimple* builds upon the previously developed Imperial College Deterministic Malaria Model [8] (see Section A of S2 File).

### Workflow

The workflow of *malariasimple* is designed to closely mirror that of *malariasimulation* allowing users to fluidly switch between the two models according to their research needs. To generate a simulation, users first define the desired scenario with regards to factors such as transmission intensity, seasonality, and intervention coverage, using helper functions that configure the relevant model parameters. These parameters are then passed into the main model which is written using odin2 [9], a domain-specific language in R which compiles to C++ for efficient simulation and provides support for parallel processing. Users can then work with the resultant time series data frame, or create more customised outputs using post-processing helper functions.

### Model structure

The malaria parasite is transmitted between humans by Anopheline mosquitoes, and as such the model consists of coupled human and mosquito sub-models*.* The mosquito life-cycle is subdivided into eggs and early larval instar stage, late instar lavae, pupae and adults, with adult mosquitos occupying susceptible, latent, or infectious states. Mosquito population density is regulated by density-dependent larval mortality, with full details presented in [2]. Immunity processes include maternally acquired immunity, which decays with age, and exposure-driven immunity, which evolves dynamically in response to an individual’s infection history. This paper focuses primarily on the human sub-model and implementation of interventions, where the main simplifications and computational efficiencies of *malariasimple* have been introduced.

**Fig 1:**
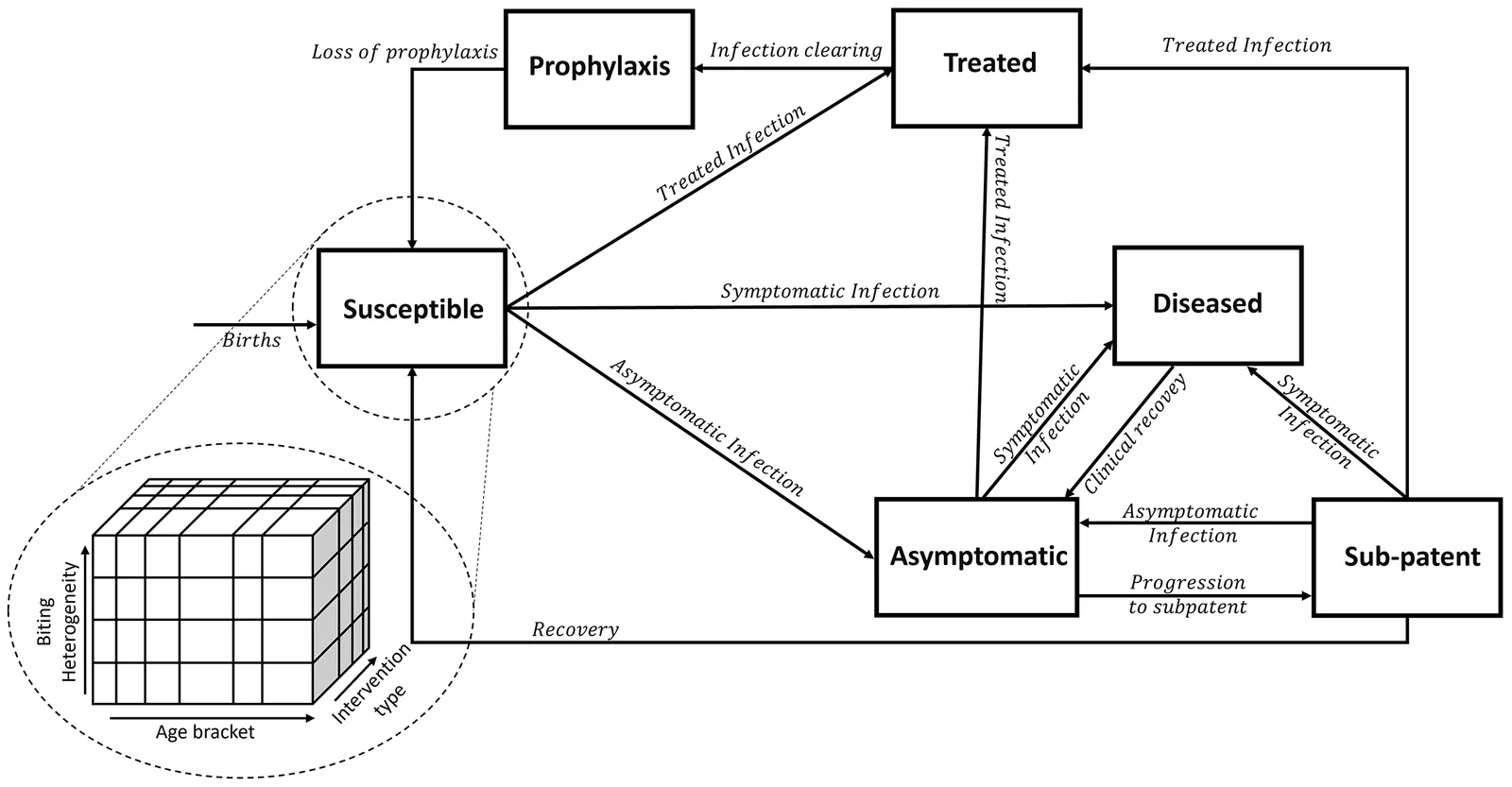
Schematic of basic model structure. Death and aging occur within all six disease states, and are omitted here for clarity. The human population is stratified by age, their susceptibility to receiving mosquito bites (biting heterogeneity), and the type of intervention they receive. These strata are illustrated for susceptible individuals but exist in all disease states.

The human population in *malariasimple* is divided into six disease states (Fig 1): susceptible, diseased (symptomatic), asymptomatic infection, sub-patent infection, treated infection, and prophylactically protected. Individuals move between human disease states according to the mosquito-derived force of infection, the probability that infection causes clinical disease, treatment coverage, and fixed recovery or prophylaxis-loss rates. Infectious bites first pass through a latent period before entering blood-stage infection. Acquired immunity, which depends on age and cumulative exposure, reduces the probability of clinical disease and shifts infections toward asymptomatic and sub-patent states. In turn, infected humans contribute to mosquito infection at rates that depend on their infection state, thereby coupling the human and mosquito sub-models.

To capture population-level variability, each disease state is further stratified along three dimensions: age, biting heterogeneity, and intervention group. We stratify by age because cumulative exposure drives the gradual acquisition of immunity, meaning older individuals typically experience lower infection risk and milder disease. It also captures variation in mosquito biting rates, which increase with body size resulting in younger children receiving fewer bites. Moreover, it can be useful to target certain interventions to specific age groups, making age stratification necessary to accurately represent their effects on transmission dynamics.

To capture additional heterogeneity in exposure beyond age, *malariasimple* assigns relative biting rates according to a log-normal distribution. This representation reflects variation in human attractiveness to mosquitoes and behavioural differences affecting exposure. The resulting heterogeneity generates positive feedback in transmission: individuals who are bitten more frequently are both more likely to acquire infection and, if infected, transmit it to mosquitoes, while those bitten less often are less likely to be infected and maintain lower levels of immunity.

Finally, the population is divided by intervention group to account for heterogeneity in exposure and immunity arising from control measures. *malariasimple* currently models the two most widely used control interventions: the use of insecticide treated nets (ITNs) and seasonal malaria chemoprevention (SMC). This stratification allows the model to represent within-population differences in transmission dynamics and to capture potential rebound effects following the introduction or withdrawal of interventions.

*malariasimple* includes a small number of tuneable numerical configuration settings that control the trade-off between runtime and approximation accuracy. These include the number of time steps per day, the number of biting heterogeneity groups, the stratifications of human ages, and the number of sub-compartments used to approximate the latent delay between exposure and blood-stage infection (see vignette *model_introduction.Rmd* for further details). Increasing the number of time steps or compartments increases the numerical resolution of the model and accuracy compared to the individual-based malariasimulation model, but increases runtime. All results presented in this paper were generated using the default model configuration and should therefore be interpreted as conditional on this.

The aim is to provide two options for the malaria transmission model: one with deterministic human transitions and one with stochastic human transitions. The deterministic option is intended to run quickly and produce smooth trajectories, making it ideal for exploring baseline dynamics, parameter sensitivity, and teaching examples.

In the stochastic formulation, transitions between human compartments are modelled as probabilistic processes. For each sub-compartment and time step, the total number of individuals leaving is first sampled from a binomial distribution, with probability determined by the combined transition rates. These individuals are then distributed across the possible destination compartments using a multinomial distribution in proportion to their respective transition rates. This approach ensures that competing risks are handled explicitly and that population counts are conserved. The stochastic formulation therefore introduces demographic variability into the model while retaining the same underlying structure and parameters as the deterministic version. In large populations (for example, over 100,000), stochastic simulations begin to closely approximate the deterministic solution, whereas in smaller populations or low-transmission settings they capture variability and extinction dynamics that are not represented in the deterministic model.

### Model initialisation

To reduce the need for a model initialisation burn-in period, simulations are initialised at endemic equilibrium using analytical solutions detailed in Griffin 2016 [10]. These solutions provide equilibrium conditions for a given treatment coverage and baseline entomological inoculation rate (EIR) in the absence of any time-varying external forcing. The resulting stratified infection states and immunity levels are used as initial conditions. When including interventions in the model, simulations are run forward from their time of introduction, allowing the system to evolve from this baseline equilibrium.

## Intervention processes

In addition to treatment therapies, two further types of control interventions are implemented in *malariasimple*: insecticide-treated nets (ITNs) and seasonal malaria chemoprevention (SMC). Both are adapted from the formulations used in *malariasimulation*, but restructured into a reduced compartmental framework that allows the effects of coverage, decay, and timing to be represented efficiently while maintaining consistency with the full model. Full mathematical formulations and parameter definitions for treatment therapies, ITNs and SMC are provided in Section C of S2 File.

### Insecticide-treated nets

ITNs are a core malaria control intervention that reduce human-mosquito contact by combining a physical barrier with an insecticide that both repels and kills mosquitoes. Hence, ITNs influence malaria transmission by both increasing mosquito mortality and reducing the mosquito biting rate, with both effects decaying over time after distribution. Upon encountering an ITN, a mosquito can either survive and successfully bite ($s_{ITN}$), be repelled ($r_{ITN}$), or be killed $(d_{ITN}$). These proportions are assumed to wane over time via two mechanisms:

1. Individuals stop using nets at a constant rate defined by mean retention period, δ.
2. The insecticidal killing activity of the ITN decays with time at a rate $\text{1}/\gamma_{n}$.

ITN deployment is assumed to be either though continuous distribution channels (for example, through antenatal care, the Expanded Programme for Immunization or schools) or at mass campaign events (everyone in an area receives a net on the same date), reflecting the different ITN distribution models currently in use [11]. If nets are distributed continuously, it is assumed there is a time-varying proportion of the population using and regularly replacing them, and that all nets decay equal to the mean over the retention period. If ITNs are distributed in a mass campaign it is assumed that a defined proportion of the population receives and starts using the ITN on a specified day. In this mode, the levels of decay and retention are tracked for each distribution event, and then averaged across the net-using population. Evidence indicates that the protective efficacy of ITNs is distributed across the population though a community effect, as it is thought mosquitoes feed on people randomly in the community resulting in only minor differences in malaria prevalence between users and non-users [12,13]. Therefore, for both distribution methods we make the simplifying assumption that the proportion of the population benefiting from an ITN is fixed, with time-varying ITN coverage captured by scaling $r_{ITN}$ and $d_{ITN}$ according to current users / maximum users. Full details and equations are provided in the Supporting Information Section C of S2 File. This linear approximation provides a computationally efficient way to measure the effect of a wide variety of ITN intervention scenarios.

### Seasonal malaria chemoprevention

SMC is a widely implemented intervention in regions with highly seasonal transmission where antimalarial drugs are administered to young children, regardless of their malaria status, in multiple rounds throughout the malaria season. In *malariasimple*, two broad assumptions can be made regarding how SMC treatments are distributed.

1. ‘Random’ distribution. Each individual within the defined age category has an equal chance of receiving SMC at each distribution event regardless of whether they received a dose previously. This generally leads to higher overall coverage.
2. ‘Correlated’ distribution. Assumes doses are given to the same children each round. If a child receives SMC on the first distribution event, they will continue to receive further doses unless the coverage decreases. This can be thought of as some children being ‘accessible’ by public health personnel, and others remaining ‘inaccessible’ for the duration of the simulation.

SMC reduces malaria transmission by three separate mechanisms:

1. Infection clearing effect. A specified proportion, $\alpha_{SMC}$, of currently infected children receiving SMC will be cleared of infection (i.e., they return to ‘susceptible’) following a fixed lag period $\tau_{SMC}$.
2. Prophylaxis effect. Individuals who have received SMC are assumed to have some protection against future infection. This is implemented in the model by reducing the force of infection to individuals who have received SMC such that $FOI_{SMC}[t]=FOI[t]\cdot (\text{1}-Pro_{SMC}[t])$, where $Pro_{SMC}[t]$ represents prophylactic protection at time t. This protection is highest immediately following administration and decays over time according to a Weibull cumulative distribution function characterized by shape $\kappa_{SMC}$ and scale $\lambda_{SMC}$.
3. Reduced infectivity. During the lag period between receiving treatment and full infection clearance, infected individuals are assumed to have reduced infectiousness to mosquitoes. Specifically, for a duration of $\tau_{SMC}$ following SMC administration, their contribution to the human-to-mosquito force of infection is scaled by a factor $\beta_{SMC}$.

## Results

We evaluate the performance of *malariasimple* by comparing its computational cost and simulated outputs with those of *malariasimulation* before demonstrating the model’s utility for inference by estimating posterior distributions for two key parameters using MCMC parameter estimation. Our analysis focuses on comparison with *malariasimulation* rather than direct observational data as *malariasimple* is intended as a fast, approximate counterpart to be used alongside *malariasimulation*, which is already well-established and validated in the literature (e.g., [10,14,15]). Details of methodology used to produce the outputs presented in this section as well as reproducible code containing full model parameterisations, simulations and outputs are provided in the S1 File.

### Comparison of computational cost

**Fig 2:**
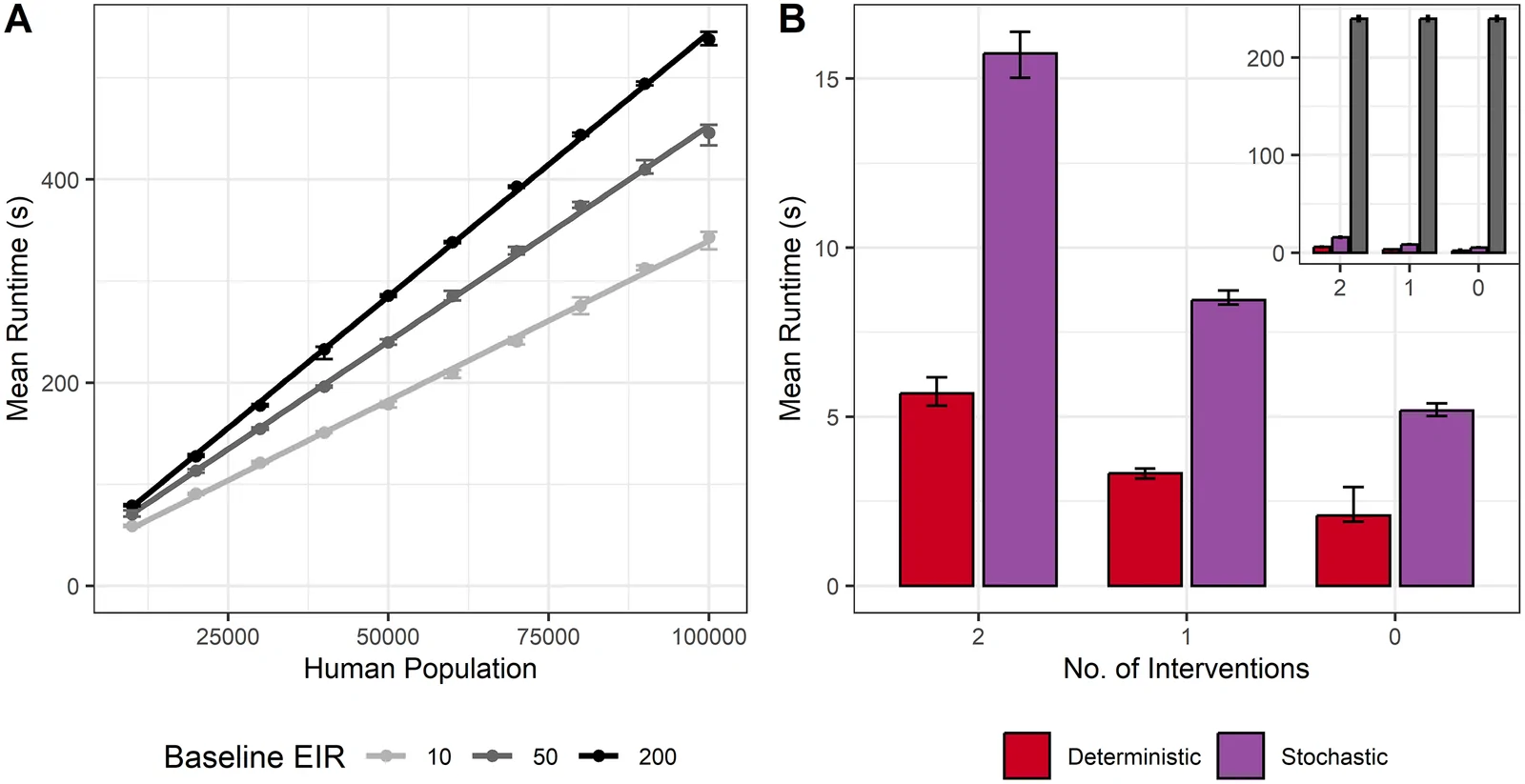
Simulation runtimes of the different models run for 20-year simulations. (A) malariasimulation: mean runtime versus human population at baseline entomological inoculation rate (EIR) of 10, 50, and 200; lines indicate best fit linear regression. (B) malariasimple: runtime by number of interventions, shown for deterministic (red bars) and stochastic (purple bars) variants using default tuneable parameters. Inset shows the same plot with the addition of a malariasimulation run with 50,000 pop and EIR = 50 for comparison (grey bars). All runs were executed on the Imperial College High Performance Cluster for consistency, however similar runtimes may be expected on a standard laptop computer. For all panels we report the mean of 20 replicates, error bars denote the range.

*malariasimulation* runtimes increased approximately linearly with population size at each EIR, ranging from a mean of 59.3 s (EIR = 10; 10,000 people) to 538.0 s (EIR = 200; 100,000 people) (Fig 2A). In comparison, deterministic *malariasimple* runs were substantially faster, from 2.1 s (no interventions) to 5.7 s (ITN + SMC) (Fig 2B). Running *malariasimple* stochastically incurred additional computational cost that increased with the number of interventions: mean runtimes were 150%, 154%, and 177% higher than the corresponding deterministic runs for 0, 1, and 2 interventions, respectively. This non-linear rise in overheads is likely due to stochastic transitions occurring only among human transitions. As interventions subdivide the human population into more strata, the number of stochastic transition calculations grows, increasing the relative contribution of human processes to total runtime. Depending on population size, transmission intensity and interventions modelled, switching to *malariasimple* reduced compute time by 90.2% (low transmission, low population, two interventions) up to 99.6% (high transmission, high population, no interventions) and 73.0% to 99.1% by switching to the stochastic version.

While *malariasimple* offers substantial improvements in computational efficiency, its performance gains diminish as more interventions are included. Each new intervention doubles the number of possible intervention combinations, since all potential overlaps (including no intervention) must be represented, leading to an exponential increase in the number of strata (${\text{2}}^{N}$ for N interventions). Beyond two simultaneous interventions, this growth begins to offset the model’s computational advantage, as the increasing number of human sub-compartments raises the overall runtime.

### Comparison of key model outputs

**Fig 3:**
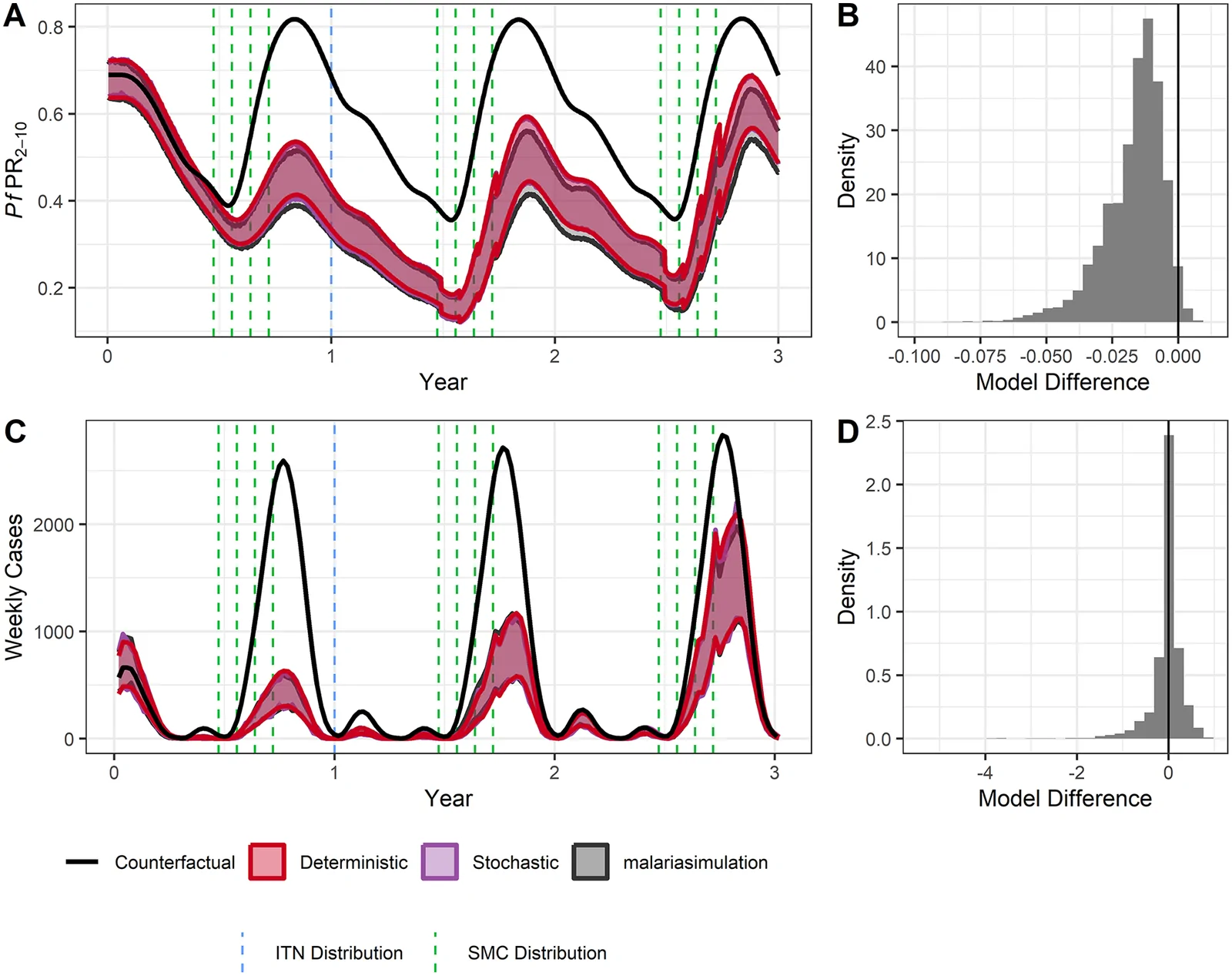
Comparison of malariasimple and malariasimulation simulation outputs for a moderate transmission intensity scenario (baseline EIR = 50) with medium sized population (N = 50,000). A and C present time series simulations of parasite prevalence in children aged 2-10, and all-age weekly cases, respectively. Ribbons represent the 90% percent credible interval for the different models. The black line denotes a counterfactual scenario produced by the deterministic malariasimple model where no interventions are introduced, the timing of which is denoted by the vertical dashed lines, for insecticide treated nets (ITN, blue) and rounds of seasonal malaria chemoprevention (SMC, green). B and D are histograms of point-wise absolute differences across all time points between stochastic malariasimulation runs and deterministic malariasimple runs such that compared outputs had identical parameter inputs. Positive values indicate higher estimates for malariasimulation.

We compared both the deterministic and stochastic versions of *malariasimple* with *malariasimulation* under an identical illustrative seasonal-transmission scenario, while propagating parameter uncertainty through repeated simulations using parameter sets sampled from previously estimated posterior distributions [14]. From Fig 3, it is clear that *malariasimple* outputs closely match those of *malariasimulation.* Across the time series of both parasite prevalence (Fig 3A) and weekly cases (Fig 3C), the 90% credible intervals for all three models at moderate transmission intensity show substantial overlap at all time points, particularly for weekly cases. This adherence to the individual model is also seen in high- and low- transmission scenarios, although with greatest similarities occurring at lower transmission intensities (see Fig B in S1 File for an expanded plot). In the context of parameter uncertainty (see section B in S1 File), our results suggest that differences between the outputs of the two models may be largely considered negligible, particularly at lower transmission intensities. Moreover, the spread of outputs is very similar across models, indicating that most of the variation arises from parameter uncertainty rather than from stochastic processes in these scenarios. Consequently, for medium- and high-population scenarios, the deterministic version of *malariasimple* offers a useful and computationally efficient additional tool for certain applications.

Direct model differences, independent of parameter variation, are illustrated in the difference histograms (Fig 3B,3D) which compare *malariasimulation* and deterministic *malariasimple* outputs from runs with identical parameter inputs. This demonstrates that, the majority of the time, *malariasimple* produces a slightly higher prevalence estimate than *malariasimulation*. In the presence of non-linear dynamics, deterministic or stochastic compartmental models may diverge slightly from their individual-based counterparts, as population averaging can smooth over heterogeneity and stochastic effects that influence transmission at the individual level. One likely source of this difference arises from how the two models represent loss of interventions. In *malariasimple*, we approximate loss of nets by linearly reducing the effectiveness of each net, proportional to net loss, for the entire ITN compartment [See Section C of S2 File for more details]. For example, if 100% of the population receive nets and 40% stop using them, *malariasimple* approximates this by assuming 100% of the population have nets which are 60% effective. In contrast, *malariasimulation* models 60% of the population with fully effective nets (ignoring insecticide decay), and the remaining 40% with zero direct protection. This same linear approximation is applied to time-varying coverage of both SMC and ITN interventions: *malariasimple* fixes the size of each intervention compartment to the maximum coverage experienced during the simulation period and linearly reduces effectiveness during periods of lower coverage. Because the relationships between exposure, immunity, and infection are non-linear, this introduces minor unavoidable discrepancies, particularly in prevalence estimates as this metric includes asymptomatic cases (unlike clinical incidence) which are strongly influenced by the non-linear interaction between exposure and immunity. Nevertheless, these differences are very small relative to the magnitude of the effect size and the parameter uncertainty explored.

The use of an individual-based model remains particularly valuable in low-population or elimination settings, where stochastic effects and individual heterogeneity play a dominant role in shaping transmission dynamics. In these contexts, model runtimes are naturally shorter, and the additional realism of an individual-based approach can provide important insights that a compartmental approximation may miss. Consequently, *malariasimple* may not always be the most appropriate tool in such scenarios. To ensure an acceptable level of accuracy for a given scenario and research objective, we recommend that new simulation experiments be benchmarked against equivalent *malariasimulation* runs to confirm that the simplified model provides an adequate approximation.

### Comparison of intervention effectiveness

**Fig 4:**
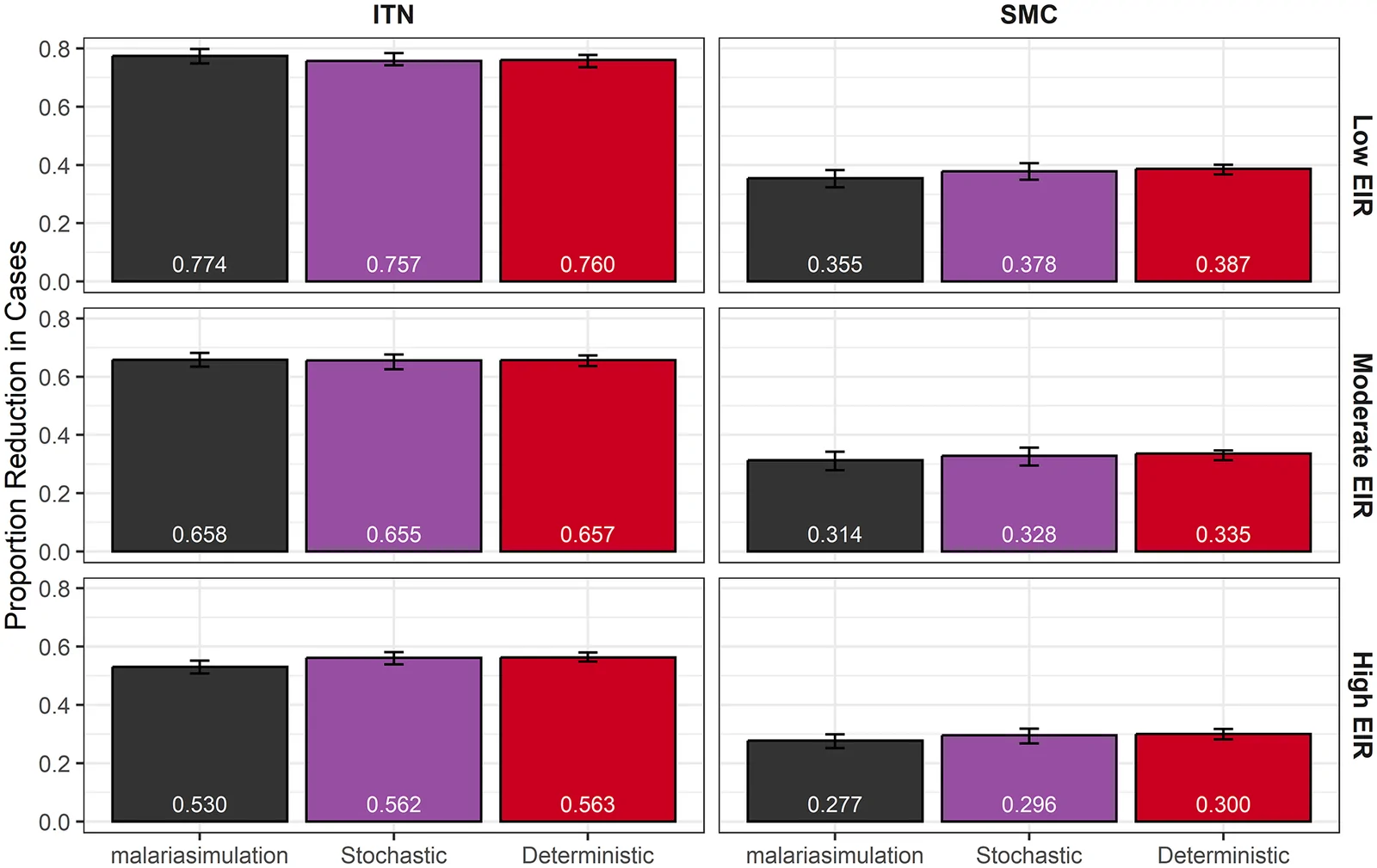
Comparison of effectiveness of interventions across the different model versions. Bars represent proportional reduction in cases over a three-year period following introduction of each intervention compared to a counterfactual scenario with no interventions. Malariasimulation is represented by dark grey, stochastic and deterministic malariasimple runs are represented by purple and red respectively. Low, moderate and high entomological inoculation rate (EIR) refer to baseline EIRs of 10, 50, and 200 respectively. Error bars show 90% credible interval for each reduction. The number inside each bar give the mean proportion reduction to 3 decimal places.

*malariasimple* (both stochastic and deterministic) and *malariasimulation* produced highly consistent estimates of intervention effectiveness, with mean differences within four percentage points (Fig 4). These differences generally fell within each model’s credible intervals and can therefore be considered negligible. All models agreed on the qualitative trends: ITNs achieved substantially greater reductions in malaria cases than SMC, and intervention effectiveness declined with increasing baseline transmission intensity (EIR).

### Example use case

**Fig 5:**
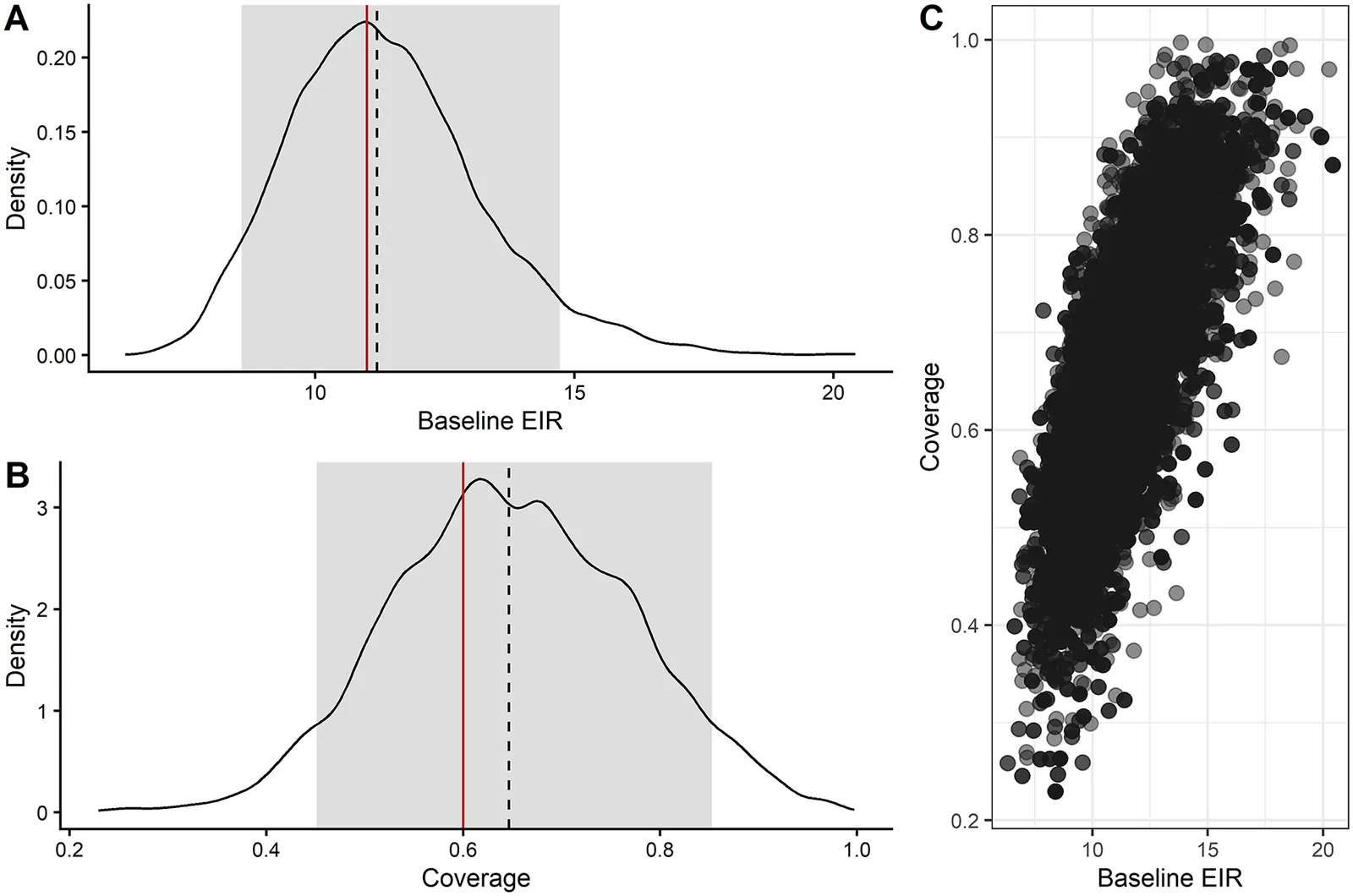
Posterior parameter estimates from the MCMC analysis. **(A, B)** Posterior density distributions for baseline EIR and ITN coverage, respectively. The black dashed line indicates the median of the posterior samples, while the red line marks the true parameter value used to generate the synthetic data. The grey box represents the width of the 90% confidence interval **(C)** Joint posterior samples showing the correlation between baseline EIR and ITN coverage.

**Fig 6:**
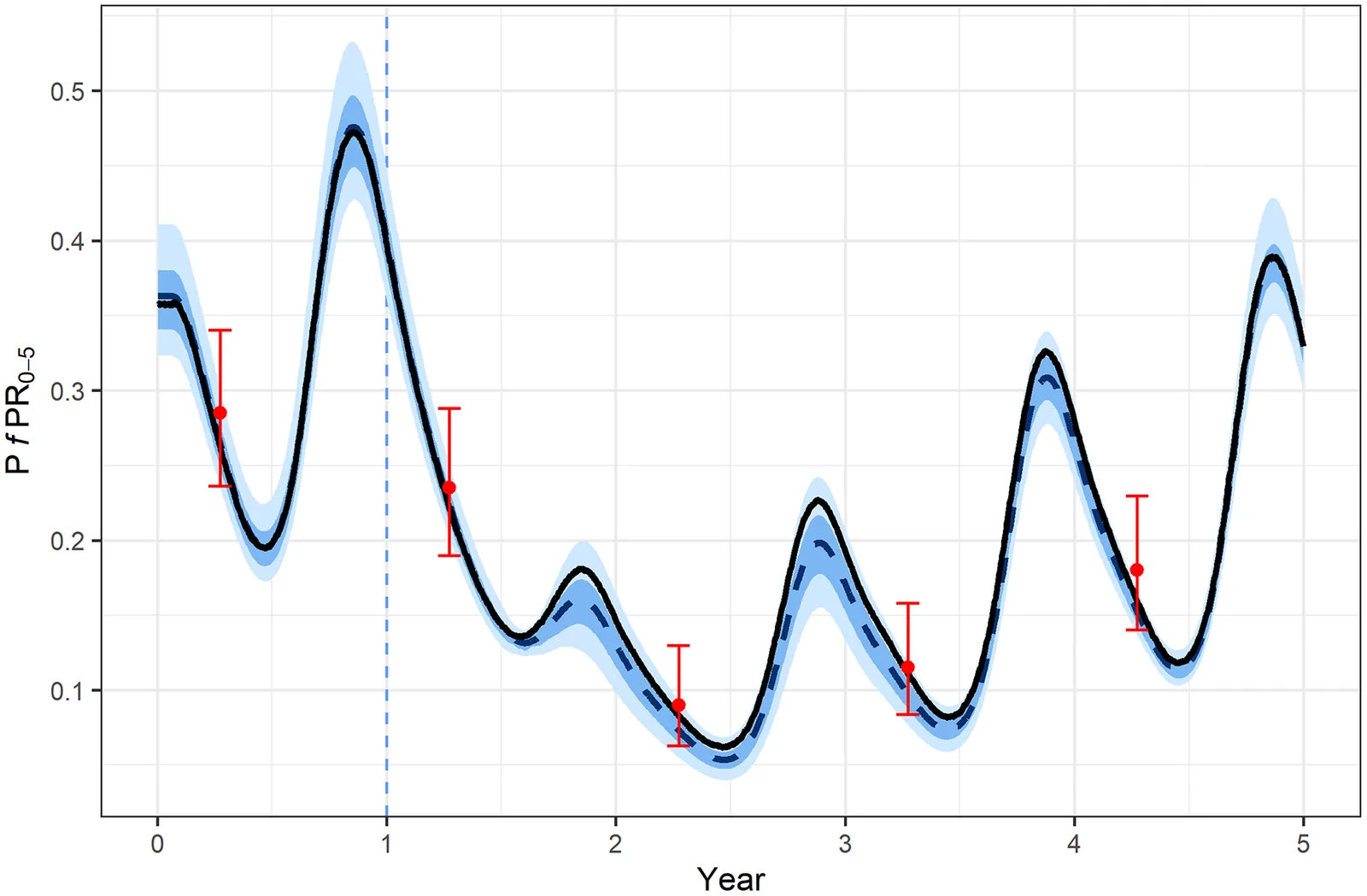
Observed data and posterior predictions of malaria prevalence over time for children under 5. The black line shows the true population prevalence, with synthetic data points (red) and associated 90% credible intervals. The model posterior is shown in blue, with the dark blue dashed line representing the posterior median and the shaded regions indicating the 50% (medium blue) and 90% (light blue) credible intervals. The vertical dashed blue line indicates the timing of the ITN distribution.

The practical usefulness of *malariasimple* for Bayesian parameter inference is illustrated in Figs 5 and 6. Fig 5A and 5B show the posterior distribution for Baseline EIR and ITN coverage, respectively. The shaded region indicates the 90% credible interval, which ranges from 8.6 to 14.7 for baseline EIR suggesting strong confidence that the region experiences low transmission. In contrast, the 90% credible interval for ITN coverage spans 0.45 to 0.85, reflecting greater uncertainty due to the imprecision in the prevalence estimates and the complicated relationship between EIR, ITN use and malaria prevalence, meaning many plausible parameter combinations can explain the observations.

The dependence between the two parameters is evident in Fig 5C where the joint posterior samples show a strong positive correlation: lower ITN coverage values are more consistent with lower EIR and vice versa, since the parameters exert opposing effects on malaria prevalence. Given the high uncertainty inherent in most malaria surveillance data, the ability to quantify and interpret parameter uncertainty is critical to represent what the data can truly inform about local transmission dynamics.

This uncertainty is naturally propagated into the model outputs, as shown in Fig 6, which presents the posterior prediction of true prevalence. The true prevalence lies largely within the 50% credible interval and entirely within the 90% interval, demonstrating that, in this example, the model effectively captures both the mean dynamics and the uncertainty around them. Moreover, the posterior distribution provides credible intervals for prevalence at any point in time, offering a probabilistic assessment of how well the fitted model represents reality.

## Availability and future directions

The model code presented in this paper is freely available for public use [github.com/mrc-ide/malariasimple], with accompanying vignettes that guide users through the model structure and its applications. It has been written and documented with the intention of being intuitive and interpretable for users with basic knowledge of infectious disease modelling and R programming, and is designed as a flexible framework that can be adapted to specific research questions and settings. We therefore intend *malariasimple* not only as a tool for the applications presented here, but also as a model structure that users can extend for their own research needs, including by adding new interventions or alternative epidemiological processes. However, users should be aware that altering model structure and parameterisation could generate misleading results as many of the parameters (outlined in [14]) were fit in parallel and are therefore conditional on each other and the underlying structural assumptions in the model. As with other models within the framework, *malariasimple* is under active development, and this work represents its first iteration. Future developments will focus on expanding the intervention portfolio to include indoor residual spraying and vaccination, enhancing flexibility, and integrating climate sensitivity.

In conclusion, *malariasimple* offers a practical and efficient extension to the Imperial College Malaria Model framework, bridging the gap between computational efficiency and epidemiological realism. By retaining the essential epidemiological processes of *malariasimulation* while substantially reducing runtime, *malariasimple* enables researchers to perform Bayesian inference, explore parameter uncertainty, and run fast, flexible simulations suitable for both research and policy applications.

## Supporting information

S1 File Methods and additional plots. (DOCX)

S2 File Transmission model. (DOCX)
