# Supplementary material for: malariasimple: An R package for fast simulations of malaria transmission
Source: PLoS Comput Biol. 2026 Sep 1;22(9):e1013687. doi: 10.1371/journal.pcbi.1013687 (PMC13561361; doi:10.1371/journal.pcbi.1013687)
Supplement: Supplementary file 1 [file pcbi.1013687.s001.docx]

## S1 – Methods and Additional Plots

For full parameterisation of all simulations and plots presented in the paper, see the accompanying reproducible R code file *Shackleton26_simulations.R* available from the GitHub repository [[github.com/DebbieShack/malariasimple_paper](https://github.com/DebbieShack/malariasimple_paper)].

### **A. Comparison of Computational Cost**

In this section, we compare the runtime of simulations from the two models. *malariasimulation* is sensitive to both human population size and transmission intensity (driven by baseline EIR) because immunity and disease transitions are updated per individual and higher EIRs generate more infection events. By contrast, *malariasimple* updates compartment totals each step and is therefore insensitive to population size and baseline EIR; its runtime is driven instead by the number of interventions (which increase the number of sub-compartments) and by whether stochastic binomial sampling is used.

To measure and compare the computational costs of a variety of scenarios, *malariasimulation* was evaluated for populations from 10,000 to 100,000 (increments of 10,000) at high, medium and low transmission scenarios (baseline EIR = 10, 50, and 200) . *malariasimple* was run under no intervention, ITN-only, and ITN+SMC scenarios, for both deterministic and stochastic variants (Figure 2B). Each simulation represented a 20-year seasonal simulation and was repeated for 20 replicates on the Imperial College London High Performance Cluster for consistency.

### **B. Comparison of key model outputs**

We simulated identical illustrative scenarios using *malariasimulation* and *malariasimple* to compare key model outputs. This scenario represents a highly seasonal setting of 50,000 population with a single ITN distribution on day 100 at 60% coverage, and SMC administered to children under 5 in four annual doses from year 2 onwards, timed to peak transmission, with coverage increasing linearly from 30% to 60%. We focus on two programmatically relevant outputs: Plasmodium falciparum prevalence among children under five (as determined by microscopy or rapid diagnostic test), the metric routinely used in Demographic and Health Surveys (DHS) to track transmission intensity, and weekly all-age clinical incidence, which reflects symptomatic burden and health-service demand. Parameter uncertainty was incorporated by running each of the three simulations (*malariasimulation* and *malariasimple*, deterministic and stochastic) 20 times, with parameters randomly sampled from their respective posterior distributions for each run. These posterior distributions were obtained through Bayesian Markov chain Monte Carlo fitting of malariasimulation to age-stratified prevalence, incidence, and infectivity data from 23 African field sites, as described in (1).

### **C. Comparison of intervention effectiveness**

A common application of disease transmission models is to evaluate the relative effectiveness of different control interventions; it is therefore important that models produce consistent estimates of intervention impact. To estimate this, we simulated the number of cases in the three years following the introduction of each intervention estimated by each model. Simulations were run for three years following intervention introduction, assuming 50% population coverage and using 20 repetitions with parameter values drawn from their respective posterior distributions (as described in Section B). For the SMC scenario, four annual treatment rounds were applied during the peak transmission season. We define effectiveness as the proportional reduction in malaria cases following the independent introduction of each intervention compared with a counterfactual scenario with no intervention.

### **D. Example Use Case**

To illustrate the practical application of malariasimple, we designed a synthetic scenario to fit malaria prevalence data following the mass distribution of ITNs in a representative, highly seasonal West African setting with a large population (N = 250,000) and low transmission (baseline EIR = 11) (see <https://github.com/DebbieShack/malariasimple_paper> for full parameterisation). The characteristics of the site were assumed to be known, but the baseline transmission intensity of the site (EIR) and the proportion of the population using an ITN following the mass campaign were treated as unknown. Using the deterministic version of malariasimple within an MCMC framework, we estimated posterior distributions for these two unknown parameters. The synthetic prevalence data (total and positive tests from randomly sampled children under 5) were randomly generated from a binomial distribution with sample sizes comparable to that seen in the DHS surveys conducted in the region to ensure realism.

MCMC sampling was performed using the adaptive random walk sampler implemented in the monty R package (2). Uninformative priors were applied: a uniform (0, 1) prior for ITN coverage and a Gamma(1.5, 0.2) prior for baseline EIR. Each of four chains was initialised with a sample from the prior and run for 5,000 iterations with the first 500 iterations discarded as ‘burn-in’. Convergence was confirmed by visual inspection of the posterior trace plot (Fig A) and through a low scale reduction factor (Rhat = 1.01).

*Fig A - MCMC traceplot for synthetic use case scenario. Top panel shows posterior estimates of the baseline entomological inoculation rate (EIR) whist the bottom panel shows ITN coverage immediately following mass distribution campaign.*

*Fig B - Comparison of malariasimple and malariasimulation simulation outputs for low (A-D) and moderate (E-H) and high (I-L) transmission intensity scenarios (baseline EIR of 1, 50 and 200 respectively). A, E, and I present time series simulations of parasite prevalence in children aged 2-10; C, G, and K present all-age weekly cases. Ribbons represent the 90% percent credible interval for the different models. The black line denotes a counterfactual scenario produced by the deterministic malariasimple model where no interventions are introduced, the timing of which is denoted by the vertical dashed lines, be it insecticide treated nets (ITN, blue) or rounds of seasonal malaria chemoprevention (SMC, green). Histograms of point-wise absolute differences across all time points between stochastic malariasimulation runs and deterministic malariasimple runs such that compared outputs had identical parameter inputs are given for prevalence (B, F, J) and weekly cases (D, H, L) outputs. Positive values indicate higher estimates for malariasimulation.*

## References

1.	Griffin JT, Ferguson NM, Ghani AC. Estimates of the changing age-burden of Plasmodium falciparum malaria disease in sub-Saharan Africa. Nat Commun. 2014 Feb 11;5. doi:10.1038/ncomms4136 PubMed PMID: 24518518.

2.	Fitzjohn R, Hinsley W, Knock E, Baguelin M. monty: Monte Carlo Models. https://github.com/mrc-ide/monty. 2025.

Click or tap here to enter text.
