# Supplementary material for: malariasimple: An R package for fast simulations of malaria transmission
Source: PLoS Comput Biol. 2026 Sep 1;22(9):e1013687. doi: 10.1371/journal.pcbi.1013687 (PMC13561361; doi:10.1371/journal.pcbi.1013687)
Supplement: Supplementary file 2 [file pcbi.1013687.s002.docx]

# S2 - Transmission Model

## **A**. Relationship between ICDMM, malariasimple, and malariasimulation

The Imperial College Deterministic Malaria Model (ICDMM) [mrc-ide.github.io/deterministic-malaria-model] is a continuous-time compartmental model formulated using ordinary differential equations and developed as a simplified approximation to the individual-based model *malariasimulation*. *malariasimple* builds upon the conceptual framework of ICDMM but extends it in several key ways. First, it introduces a discrete-time formulation using difference equations allowing greater flexibility in implementation. Second, it includes both deterministic and stochastic variants, enabling users to capture either smooth average dynamics or random variation in transmission. Third, it substantially expands the representation of interventions, which are only treated in a limited way in ICDMM. Finally, the model interface and workflow are designed to closely mirror *malariasimulation*, allowing users to move seamlessly between the fast compartmental approximation and the more detailed individual-based model.

## B. Human Disease Transitions

In this section we provide further details regarding the transitions between human disease states. The names of the individual processes are identical to those given in Fig 1 of the main text. Descriptions of model parameters are given in Table A. Throughout this section $i$, $j$, and $k$ refer to 3d array positions within each state, referencing age, biting heterogeneity, and intervention category stratifications respectively.

The six human compartments are intended to represent epidemiologically distinct infection and treatment states (See Fig 1 in main text). Within these, we explicitly distinguish four infection states: *Diseased* (but untreated), *Treated* (clinical disease that received prompt and effective treatment), *Asymptomatic*, and *Sub-patent*. Each state differs in their onward infectivity to mosquitoes, their probability of being detected by surveillance (and hence their contribution to measured malaria prevalence), and whether they are classified as a clinical ‘case’ when comparing model outputs with malaria case datasets (1).

### B.1. Demographics

Death of humans due to malaria are considered a sufficiently small proportion of total deaths as to be ignored, so the death rate of all disease states ($X$) is assumed to be equal.

$$Deat{h[i,j,k]}_{X}=\eta$$

The death rate, $\eta$ is user defined and assumed constant across age groups, intervention compartments, biting heterogeneity compartments and disease states. $dt$ represents the width of one time step.

$$Births\left[ 1,j,k \right]=H\cdot\eta\cdot Bg\left[ j \right]\cdot Ig\left[ k \right]$$

Birth and death rate $\eta$ are assumed equal to provide a stable human population. $Bg[j]$ and $Ig\left[ k \right]$ represent the population proportion in each biting and intervention division respectively. All births are added to the youngest age group and are divided proportionally across bite and intervention divisions:

$$Aging\left[ i,j,k \right]_{X}=\frac{1}{Age\left[ i+1 \right]-Age[i\boldsymbol{]}}$$

Aging occurs across all interventions, bite divisions, and disease states $X$. The rate of aging is set to the inverse of the width of each age category. Aging in the oldest age division is set to zero.

### B.2 Infection Processes

The infection process is governed by the following equations which match the graphical representation seen in Figure 1 of the main text,

$$Treated Infection\left[ i,j,k \right]=\Lambda\left[ i,j,k \right]\cdot\phi\left[ i,j,k \right]\cdot f_{T}$$

$$Symptomatic Infection\left[ i,j,k \right] =\Lambda\left[ i,j,k \right]\cdot\phi\left[ i,j,k \right]\cdot(1-f_{T})$$

$$Asymptomatic Infection\left[ i,j,k \right]=\Lambda\left[ i,j,k \right]\cdot(1-\phi\left[ i,j,k \right])$$

where $\phi$ and $f_{T}$ represent probability of clinical disease and the proportion of cases treated promptly respectively.

The force of infection $\Lambda$ depends on the entomological inoculation rate (EIR) and the level of infection-blocking immunity $b$ (see (1) for further details). There is a delay between EIR and $\Lambda$ due to the latent liver stage of infection such that:

$$\Lambda_{0}\left[ i,j,k \right]=EIR\left[ i,j,k \right]\cdot b[i,j,k]$$

Where $\Lambda_{0}$ represents the force of infection with no delay. Since the model runs as a Markov-chain and as such is memoryless, we implement the delay using a chain of $n_{\Lambda}$ compartments which progress sequentially through $n_{\Lambda}$ intermediate stages ($\Lambda_{0},\Lambda_{1},\ldots,\Lambda_{n_{\Lambda}}$) each with transition rate $n_{\Lambda}/dE$, where $dE$ is the length of the latent period. The final output $\Lambda_{n_{\Lambda}}$ represents the effective force of infection, $\Lambda$, acting on the population. This construction results in the time spent in latent period following an Erlang distribution with mean $dE$. As $n_{\Lambda} \to\infty$ the distribution becomes narrower and approximates a fixed delay of $dE$, however higher values come at the cost of increased runtime. The default value given in *malariasimple,* and that used in the simulations in this paper is $n_{\Lambda}=10$.

### B.3. Recovery

Remaining processes are defined by single user-defined static rates applied equally across all sub-compartments.

$$Clinical recovery[i,j,k]=r_{D}$$

$$Progression to subpatent[i,j,k]=r_{A}$$

$$Infection Clearing[i,j,k]=r_{T}$$

$$Loss of prophylaxis[i,j,k]=r_{P}$$

| **Parameter** | **Parameter name in model code** | **Definition** |
| --- | --- | --- |
| $Age[]$ | age_vector | Vector denoting maximum age for each age bracket |
| $Bg[]$ | het_wt[] | Vector denoting proportion of population in each bite division |
| $Ig[]$ | cov[] | Vector denoting proportion of population in each intervention type division |
| $H$ | H | Total human population size |
| $\phi$ | phi | Probability of clinical disease |
| $f_{T}$ | ft | Proportion of cases treated promptly |
| $\Lambda$ | FOI | Force of infection from humans to mosquitoes |
| $dE$ | dE | Mean human latent period (days) |
| $n_{\Lambda}$ | lag_rates | Number of delay sub-compartments within $\Lambda$ |
| $r_{D}$ | rD | Rate of recovery from clinical disease to asymptomatic infection (conditional on survival) (day^-1^) |
| $r_{A}$ | rA | Rate of recovery from asymptomatic disease (conditional on survival) (day^-1^) |
| $r_{T}$ | rT | Rate at which treated individuals become no longer infectious and gain prophylactic protection (day^-1^) |
| $r_{P}$ | rP | Rate at which individuals lose prophylactic protection (day^-1^) |

Table A – Human disease state transition parameter definitions

## C. Control interventions

This section describes how interventions are implemented in the model. Parameter definitions are provided in Tables B and C.

### C.1. Defining Treatment Therapies

Malaria treatment is represented explicitly in the model through the inclusion of the *Treated* ($T$) and *Prophylactically Protected* ($P$) states. A user-defined proportion of cases, $f_{T}$, is transferred to the *Treated* state, from which individuals then move to the $P$ state before returning to the *Susceptible* ($S$) state (see Figure 1 in the main text). Individuals in both $T$and $P$ are assumed to be protected from further infection. The time spent in each state is assumed to follow an exponential distribution, such that individuals leave the $T$ and $P$ states at constant rates $r_{T}$ and $r_{P}$, respectively.

Individuals in the $T$ state are assumed to remain infected, but to have reduced infectivity to mosquitoes relative to untreated diseased individuals. Their contribution to the human-to-mosquito force of infection ($FOI_{v}$) is represented by the term $c_{T}T[i,j,k]$, where generally $c_{T}<c_{D}$. Here, $c_{T}$and $c_{D}$are compartment-specific per-capita infectiousness parameters that scale the contribution of individuals in $T$ and and *Diseased* $(D)$ states to $FOI_{v}$ respectively.

### C.2. Incorporating Insecticide treated nets (ITNs)

As in (1), ITNs influence transmission parameters in the model via two variables.

- $d_{ITN}$ which describes the proportion of mosquitoes who die upon encountering an ITN
- $r_{ITN}$ which describes the proportion of mosquitoes repelled

These variables in turn influence the time between bites. How these variables are estimated depends on the type of ITN in use, the level of insecticide resistance in the mosquito population and the user assumptions regarding ITN distribution contexts.

#### Continuous Distribution

The simplest transmission mode assumes that ITNs are distributed continuously to the population. In this scenario, a given, time-varying proportion of the population is assumed to use a single type of ITN which they regularly replace according to a given replacement period. Hence, in large populations this can be approximated by assuming the entire ITN-using population is using an ITN with insecticide decay equal to the mean level of decay over the retention period. This enables us to simply interpolate between points to create an estimated daily time series.

As in (1), we assume insecticidal activity of the ITN decays exponentially with time according to mean lifetime $\gamma_{n}$. In the continuous scenario, $d_{ITN}$and $r_{ITN}$ are taken as the average value across the retention period $\delta$.

$$decay_{mean}=\frac{1}{\delta} \int_{0}^{\delta} e^{-t\cdot\frac{1}{\gamma_{n}}} dt$$

$d_{ITN}$ is then simply:

$$d_{ITN}=d_{ITN,0}\cdot decay_{mean}$$

Repellency, $r_{ITN}$, consists of two factors:

a) A permanent net-fabric contribution of $r_{nm}$
b) An insecticide contribution $r_{n}-r_{nm}$ that decays over time

Hence,

$$r_{ITN}=r_{nm}+\left[ \left( r_{n}-r_{nm} \right)\cdot decay_{mean} \right]$$

#### Discrete Distribution

In many countries ITNs are distributed to the population during discrete ITN distribution ‘events’ or mass campaigns. Under this scenario, it is assumed that all ITNs within the population are received during one of these events. The discrete ITN framework is more flexible compared with continuous and users may define different types of ITN for each distribution event.

##### *Coverage*

Under the discrete ITN distribution framework, overall daily ITN coverage is determined from a set of user-defined distribution events. Each event is defined by two vectors:

- $\text{T}_{\text{ITN}}[i]$: the simulation day on which ITNs from distribution event $i$are distributed, and
- $C_{\text{ITN}}[i]$: the proportion of the population receiving ITNs during event $i$.

The expected retention time of an ITN in use is denoted by $\delta$, corresponding to a constant discard rate of $1/\delta$.

A coverage matrix $M_{c_{\text{ITN}}}(t,i)$with dimensions $N_{T}\times(N_{d,\text{ITN}}+1)$is constructed, where $N_{T}$is the total number of simulation days and $N_{d,\text{ITN}}$ is the number of ITN distribution events. Each element of the matrix defines the proportion of individuals at time $t$ who are using an ITN from distribution event $i$. The final column ($i=N_{d,\text{ITN}}+1$) represents individuals not currently using any ITN, such that all rows of the matrix sum to 1.

At the start of the simulation, prior to the first distribution event, no individuals use ITNs such that all rows $t<T_{ITN}[1]$ are set to

$$(0,\ldots,0,1)$$

If a distribution occurs on day 1, then the first column is initialised to the specified coverage:

$$M_{C_{\text{ITN}}}\left( 1,1 \right)=C_{\text{ITN}}\left[ 1 \right]$$

$$M_{C_{\text{ITN}}}(1,N_{d,\text{ITN}}+1)=1-C_{\text{ITN}}[1]$$

###### Daily Updates

For each subsequent simulation day $t=2,\ldots,N_{T}$:

1. **Net discarding.** Individuals discard ITNs at a constant rate $1/\delta$, resulting in exponential decay of each ITN cohort:

$$M_{c_{\text{ITN}}}(t,i)=M_{c_{\text{ITN}}}(t-1,i)\cdot e^{-1/\delta},1\leq i\leq N_{d,\text{ITN}}$$

The final column is updated to maintain row sums equal to 1:

$$M_{c_{\text{ITN}}}(t,N_{d,\text{ITN}}+1)=1-\sum_{i=1}^{N_{d,\text{ITN}}} M_{c_{\text{ITN}}}(t,i).$$

2. **New Distributions:** If day $t$ coincides with a distribution event $\text{T}_{\text{ITN}}[i]$, then a proportion $C_{\text{ITN}}[i]$ of the population receives new ITNs:

$$M_{C_{\text{ITN}}}(t,i)\leftarrow C_{\text{ITN}}[i].$$

To ensure the population size remains constant, individuals are randomly drawn from all other groups $j\neq i$in proportion to their previous representation:

$$M_{c_{\text{ITN}}}(t,j)=M_{c_{\text{ITN}}}(t,j)-C_{\text{ITN}}[i]\cdot M_{c_{\text{ITN}}}(t,j),j\neq i,\text{ }1\leq j\leq N_{d,\text{ITN}}+1.$$

##### Overall ITN Coverage

Daily ITN coverage $c_{\text{ITN}}(t)$, is then calculated as the sum across all ITN-using columns:

$$c_{\text{ITN}}(t)=\sum_{i=1}^{N_{d,\text{ITN}}} M_{C_{\text{ITN}}}(t,i)$$

This represents the total proportion of the population currently using an ITN at time $t$.

##### ITN Effectiveness

In order to estimate key variables $d_{ITN}(t)$ and $r_{ITN}(t)$, we first track the level of insecticide decay of ITNs from each distribution group $i$ at the time $t$ in the form of a matrix $M_{decay}(t,i)$.

$$M_{decay}(t,i)= \left\{ \begin{aligned} e^{\left( t-T_{ITN}\left[ i \right] \right)\cdot\frac{1}{\gamma_{n}}} t\geq T_{ITN}[i] \\ 0 t<T_{ITN}[i] \end{aligned} \right.$$

Where $T_{ITN}$ is the vector of simulation days corresponding to ITN distribution days, and $\gamma_{n}$ is the mean lifetime of insecticide.

For each ITN distribution $i$, the time-varying values of $d_{ITN}$ and $r_{ITN}$ are calculated in matrix form as:

$$M_{d_{ITN}}(t,i)=M_{decay}(t,i)\cdot d_{ITN,0}[i]$$

$$M_{r_{ITN}}\left( t,i \right)=M_{decay}\left( t,i \right)\cdot\left[ r_{n}\left( i \right)-r_{nm}\left( i \right) \right]+r_{nm}(i)$$

To maintain computational efficiency, the model represents all ITN users within a single compartment, rather than one per distribution event. Therefore the overall mean time-varying ITN effectiveness for current net users is obtained by weighting each event’s decay-adjusted efficacy by its proportional coverage:

$$x_{ITN}\left( t \right)=\sum_{i=1}^{N_{dist}} \frac{M_{C_{ITN}}\left( t,i \right)}{\sum_{i=1}^{N_{dist}} M_{C_{ITN}}\left( t,i \right)}\cdot M_{x_{ITN}}(t,i)$$

Where $x_{ITN}$ represents either $d_{ITN}$ or $r_{ITN}$.

#### Time-varying coverage

In both the continuous and discrete distribution scenarios, it is useful be able to model ITN coverage which changes over time. This adds a complication within the compartmental framework of *malariasimple* because the size of each intervention compartment cannot vary during the simulation. To permit time-varying coverage, we set the size of the ITN compartment(s) according to the maximum coverage levels during the simulation period $max(C_{ITN}\left( t \right))$, and scale effectiveness of ITNs (via the values of $d_{ITN,eff}$ and $r_{ITN,eff}$) by the ratio of current coverage to maximum coverage.

$$x_{ITN,eff}\left( t \right)=x_{ITN}\left( t \right)\cdot\frac{c_{ITN}\left( t \right)}{\max\left( c_{ITN}\left( t \right) \right)}$$

Where again, $x_{ITN,eff}$ represents either $d_{ITN,eff}$ or $r_{ITN,eff}$.

In the current simulations it is assumed pyrethroid-only ITNs are distributed and that all mosquitoes are susceptible to this insecticide, so ITNs are working optimally.

| **Parameter** | **Parameter name in model code** | **Definition** |
| --- | --- | --- |
| $d_{ITN,0}$ | dn0 | Probability of a mosquito dying upon encounter with brand new ITN (dependent on the level of insecticide resistance in the mosquito population) |
| $r_{n}$ | rn | Probability of mosquito being repelled upon encounter with brand new ITN (dependent on the level of insecticide resistance in the mosquito population) |
| $r_{nm}$ | rnm | Probability of mosquito being repelled upon encounter with old net (without insecticide) |
| $\gamma_{n}$ | gamman | Mean lifetime of ITN insecticide (days) |
| $\delta$ | retention | Average duration people retain and sleep under an ITN (days) |
| $N_{d,ITN}$ | n_dists | Number of ITN distribution events. |
| $T_{ITN}$ | days | Vector of length $N_{d,ITN}$ denoting days on which ITN treatments are administered |
| $C_{ITN}$ | coverages | Vector of length $N_{d,ITN}$ denoting proportion of population receiving an ITN on each administration day |
| $c_{ITN}$ | daily_itn_cov | Daily vector of length $T_{ITN}$ denoting proportion of population who are using some form of ITN. |

Table B – ITN Intervention parameter definitions

### C.3 Incorporating SMCs

#### Coverage

Overall daily SMC coverage, $c_{\text{SMC}}(t)$, for the specified age groups is determined by two vectors of length $N_{d,\text{SMC}}$(the number of SMC distribution events):

- $T_{\text{SMC}}[i]$: the simulation day of each distribution event $i$
- $C_{\text{SMC}}[i]$: the proportion of SMC-age children treated during each event $i$.

The value of $c_{\text{SMC}}(t)$ also depends on whether the user has selected a ‘random’ or ‘correlated’ distribution mode.

To track the temporal dynamics of SMC coverage, a coverage matrix $M_{c_{\text{SMC}}}(t,i)$ is created with dimensions $N_{T}\times(N_{d,\text{SMC}}+1)$, where $N_{T}$ is the total number of simulation days. Each element of this matrix defines the proportion of SMC-age individuals at time $t$ whose most recent treatment was from distribution event $i$. The final column ($i=N_{d,\text{SMC}}+1$) represents individuals who have never received treatment, ensuring that each row sums to 1.

The coverage matrix $M_{c_{SMC}}(t,i)$ is initialised such that all rows $t<T_{SMC}[1]$ are set to

$$\left( 0,\ldots,0,1 \right),$$

meaning that no individuals have yet received treatment.

For each distribution event $i$ occurring on day $t=T_{SMC}[i]$:

1. The corresponding column $i$ is updated to reflect the new proportion of SMC-age children created

$$M_{c_{SMC}}\left( t,i \right)\leftarrow C_{SMC}[i]$$

2. If ‘random’ distribution is selected:

Newly treated individuals at the event are drawn proportionally from all other groups $j\neq i$, ensuring that each row continues to sum to 1.

$M_{c_{SMC}}(t,j)=M_{c_{SMC}}(t-1,j)-C_{SMC}[i]\cdot M_{C_{SMC}}[t-1,j]$, $j\neq i, (1\leq j\leq N_{d,SMC}+1)$

3. If ‘correlated’ distribution is selected:

Individuals newly treated at event $i$ are preferentially drawn from those who were previously protected.

- Let the total previously protected proportion at the previous time step be:

$$P(t-1)=\sum_{i=1}^{N_{d,SMC}} M_{C_{SMC}}(t-1,i)$$

- Each previously protected column $j\neq i$ is reduced proportionally such that their total decreases by the amount required to reach the new coverage $C_{SMC}[i]$:

$M_{c_{SMC}}\left( t,j \right)\leftarrow M_{c_{SMC}}(t-1,j)\cdot\frac{\max\left( P(t-1)-C_{SMC}\left[ i \right],0 \right)}{P(t-1)}$, $j \neq i, (1\leq j\leq N_{d,SMC})$

- If total coverage increases such that previously protected groups are fully depleted, additional individuals are drawn from the final column ($N_{d,SMC}+1)$, representing those who have never received SMC:

$M_{c_{SMC}}(t,N_{d,SMC})\leftarrow1-\sum_{i=1}^{N_{d,SMC}} M_{c_{SMC}}(t-1,i)$

4. The overall SMC coverage at time $t$ is then given by the sum across all protected columns:

$$c_{SMC}\left( t \right)=\sum_{i=1}^{N_{d,SMC}} M_{c_{SMC}}(t,i)$$

#### SMC Efficacy

1. *Infection Clearing Effect***.** The primary mechanism by which SMC influences transmission is by clearing a proportion $\alpha_{SMC}$ of infections $\tau_{SMC}$ days after each treatment event $i$. Specifically, individuals in the Disease (D), Asymptomatic (A), and Sub-patent (U) disease compartments are returned to the Susceptible (S) compartment on days $t=T_{SMC}[i]+\tau_{SMC}$

For all $t\in T_{SMC}+\tau_{SMC}$:

$$\left( D\left( t \right), A\left( T \right), U\left( t \right) \right)= D\left( t-1 \right), A\left( t-1 \right), U\left( t-1 \right)\cdot\left( 1-\alpha_{SMC,eff} \right)$$

$S\left( t \right)=S\left( t-1 \right)+\alpha_{SMC,eff}\left( t \right)\cdot(D\left( t-1 \right)+A\left( t-1 \right)+U\left( t-1 \right))$

where $\alpha_{SMC,eff}(t)$ represents the *effective* infection clearance.

To maintain a constant intervention category strata, the size of the SMC sub-compartment is set to the maximum coverage observed during the simulation $max(c_{SMC}\left( t \right))$. Varying coverage is then approximated by scaling the clearance efficacy as follows:

$$\alpha_{SMC,eff}(t)=\alpha_{SMC}\cdot\frac{c_{SMC}\left( t \right)}{max(c_{SMC}\left( t \right))}$$

2. *Prophylaxis effect.* The prophylaxis effect acts by reducing the force of infection ($FOI)$ on individuals who have recently received SMC. This is protection is described by $\rho_{SMC}(t)$, the probability that an individual is protected from infection at time $t$.

$$FOI_{SMC}\left( t \right)=FOI\left( t \right)\cdot[1-\rho_{SMC}\left( t \right)]$$

Multiple SMC distribution events are represented using a matrix formulation ($M_{\rho_{SMC}})$ to capture overlapping protection from different distributions.

The residual prophylactic effect of each SMC event is assumed to decay over time according to a Weibull cumulative distribution function with shape parameter $\kappa_{SMC}$ and scale parameter $\lambda_{SMC}$.

$$M_{\rho_{SMC}}(t,i)= \left\{ \begin{aligned} e^{-\left( \frac{\left( t-T_{SMC}\left[ i \right] \right)}{\lambda_{SMC}} \right)^{\kappa_{SMC}}} t\geq T_{SMC}[i] \\ 0 t<T_{SMC}[i] \end{aligned} \right.$$

The matrix $M_{\rho_{SMC}}(t,i)$ is then multiplied by the corresponding SMC coverage matrix $M_{c_{SMC}}(t,i)$, and summed across all distribution events to estimate the average level of protection at time $t$.

$$\rho_{SMC}(t)=\sum_{i=1}^{N_{d,SMC}} M_{\rho_{SMC}}\left( t,i \right)\cdot M_{c_{SMC}}(t,i)$$

3. *Reduced Infectivity.* During the lag period between SMC administration and infection clearance ($\tau_{SMC}$), individuals retain infection but have reduced infectivity to mosquitoes. During this period, their contribution to the human-to-mosquito FOI is reduced by a factor $\beta_{SMC}$. As with the infection clearing effect, $\beta_{SMC,eff}$ is scaled to reflect varying coverage levels.

##### $\boldsymbol{\beta}_{\boldsymbol{SMC,eff}}\left( \boldsymbol{t} \right)\boldsymbol{=}\left\{ \begin{aligned} \boldsymbol{1-}\left( \left( \frac{\boldsymbol{C}_{\boldsymbol{SMC}}\left[ \boldsymbol{i} \right]}{\max\left( \boldsymbol{c}_{\boldsymbol{SMC}}\left( \boldsymbol{t} \right) \right)} \right)\boldsymbol{\cdot}\left( \boldsymbol{1-}\boldsymbol{\beta}_{\boldsymbol{SMC}} \right) \right)\boldsymbol{,}\mathbf{for}\boldsymbol{t\in[}\boldsymbol{T}_{\boldsymbol{SMC}}\left[ \boldsymbol{i} \right]\boldsymbol{,}\boldsymbol{T}_{\boldsymbol{SMC}}\left[ \boldsymbol{i} \right]\boldsymbol{+}\boldsymbol{\tau}_{\boldsymbol{SMC}}\boldsymbol{-1]} \\ \\ \boldsymbol{1}\mathbf{otherwise.} \end{aligned} \right.$

| Parameter | Name in Model Code | Description |
| --- | --- | --- |
| $\kappa_{SMC}$ | shape_smc | Shape parameter of the Weibull distribution describing prophylactic decay |
| $\lambda_{SMC}$ | scale_smc | Scale parameter of the Weibull distribution describing prophylactic decay |
| $\alpha_{SMC}$ | drug_efficacy | Proportion of infected individuals for whom infection is cleared after SMC treatment |
| $\tau_{SMC}$ | smc_clearance_lag | Lag between receipt of treatment and infection clearance |
| $\beta_{SMC}$ | drug_rel_c | Percentage reduction in human-to-mosquito transmission probability cause by the SMC administration |
| $N_{d,SMC}$ | n_dists | Number of SMC administration events |
| $T_{SMC}$ | days | Vector of length $N_{d,SMC}$ denoting days on which SMC treatments are administered |
| $C_{SMC}$ | coverages | Vector of length $N_{d,SMC}$ denoting proportion of the age-eligible population receiving SMC treatment of each administration day |
| $c_{SMC}$ | daily_smc_cov | Daily vector of length $T_{SMC}$ denoting proportion of age-eligible population who have previously received SMC and are continuing to experience some prophylactic protection |

Table C - SMC intervention parameter definitions

## D. Stochastic Human Transitions

In the stochastic version of the model, human transitions are modelled as stochastic binomial processes such that in very large populations, the simulation will approximate the deterministic solution.

To simulate competing risks between different compartments, we first draw the total number of individuals leaving a sub-compartment from a Binomial distribution, with the probability determined by the sum of outflow rates over the time step. The destination of each individual is then sampled from a Multinomial distribution, with destination probabilities proportional to the corresponding transition rates. This ensures that outflows are stochastic realisations, rather than deterministic proportions, while also ensuring that the total number leaving a sub-compartment cannot exceed the number present at the start of the time step. In the implementation, this Multinomial allocation is represented using an equivalent sequence of conditional Binomial draws, as Multinomial draws are not directly supported in odin.dust. The indices *i*, *j*, and *k* refer to positions in the three-dimensional state arrays, corresponding to age group, biting heterogeneity group, and intervention category, respectively.

$$R_{X_{total}}[i,j,k]=\sum_{m=1}^{n_{leave}} R_{X_{m}}[i,j,k]$$

where $R_{X_{m}}$ represents the rate of the $m^{th}$ transition leaving any disease state $X$. $R_{X_{total}}$ represents the total rate of individuals leaving disease state $X$. The probability of an individual leaving state $X$ within a given timestep ($P_{X}$) is then given by,

$P_{X}\left[ i,j,k \right]=1-e^{(-{R_{X}}_{total}\left[ i,j,k \right])}$.

The total number of individuals leaving state $N_{X_{leave}}$can be sampled from a binomial distribution with $X$ trials and probability $P_{X}$.

$$N_{X_{leave}}\left[ i,j,k \right] \sim Binomial(X\left[ i,j,k \right], P_{X}\left[ i,j,k \right])$$

Once the total number of individuals leaving each sub-compartment of a disease state has been ascertained, these are then apportioned to each separate transition direction using a multinomial distribution according to their relative rates.

$$\left( N_{X\to1},\ldots,N_{X\to M} \right)\left[ i,j,k \right] \sim Multinomial\left( N_{X_{leave}}\left[ i,j,k \right], \left( \frac{R_{X_{1}}}{R_{X_{total}}}, \ldots,\frac{R_{X_{M}}}{R_{X_{total}}} \right) \right)$$

## E. Estimation of Entomological Inoculation Rate (EIR)

The EIR is calculated as,

$EIR\left[ i,j,k \right]=\frac{I_{v}\cdot\psi[i]\cdot\zeta\left[ j \right]\cdot\alpha\left[ k \right]}{\omega}$.

The biting rate is assumed lower in younger age groups due to their smaller body size. The relative biting rate at age $i$, $\psi(i)$ is given by

$$\psi(i)=1-\left( \rho{\cdot e}^{-\frac{age(i)}{a_{0}}} \right)$$

Where $(1-\rho)$ is the relative biting rate at birth compared with adults, $age(i)$ is the mean age in age group *i*, and $a_{0}$ is a scale parameter for age-dependent biting.

We also assume some humans will have consistently greater exposure to bites over their lifetime due to differences such as physiology, socio-economic status and activity patterns. Each biting group $j$ has a unique relative biting rate $\zeta(j)$ which is modelled as log-normally distributed with mean one.

$$\log\left( \zeta\right)\mathcal{\sim N}\left( -\frac{\sigma^{2}}{2},\sigma^{2} \right)\mathbb{, E}\left[ \zeta\right]=1$$

This is then discretised for each distinct biting group $j$ using Gaussian quadrature.

| **Parameter** | **Parameter name in model code** | **Description** |
| --- | --- | --- |
| $I_{v}$ | Iv | Size of infectious mosquito population |
| $\psi(i)$ | foi_age[i] | Relative biting rate in age group *i* |
| $\zeta(j)$ | rel_foi[j] | Relative biting rate in biting group *j* |
| $\alpha(k)$ | av_mosq[k] | Rate at which a mosquito takes a human blood meal in intervention group *k* |
| $\omega$ | omega | Normalising constant |

Table D - Key parameters in the calculation of EIR

# References

1.	Griffin JT, Hollingsworth TD, Okell LC, Churcher TS, White M, Hinsley W, et al. Reducing Plasmodium falciparum malaria transmission in Africa: A model-based evaluation of intervention strategies. PLoS Med. 2010 Aug;7(8). doi:10.1371/journal.pmed.1000324 PubMed PMID: 20711482.
