# Supplementary material for: malariasimple: An R package for fast simulations of malaria transmission
Source: PLoS Comput Biol. 2026 Sep 1;22(9):e1013687. doi: 10.1371/journal.pcbi.1013687 (PMC13561361; doi:10.1371/journal.pcbi.1013687)
Supplement: Supplementary file 3 [file pcbi.1013687.s003.docx]

We would like to thank all reviewers for a thorough evaluation of the manuscript which we feel is now substantially improved.

Reviewer #1

The authors developed the `malariasimple` R package, which implements a simplified, faster version of a malaria transmission model. The structure, model compartments, and modelled interventions are briefly described. The outputs of `malariasimple` are compared to those of the more complex `malariasimulation` individual-based model, showing that `malariasimple` is much faster to run while achieving similar outputs.

I believe that improving the runtime of complex models to enable parameter inference and other analyses is important, and the present work makes a valuable contribution to support such efforts.

Major concerns

**1.1** The text provides two key pieces of overview information about `malariasimple`: it is a "discrete-time compartmental model," and it is "an update to a previously developed continuous-time compartmental model known as the Imperial College Deterministic Malaria Model (ICDMM)." Although this information is helpful, further clarity and more information is needed.

Although a reference (3) is provided for ICDMM, that reference is also sparse on details. I would appreciate it if the main text briefly provided basic information such as:

Is ICDMM a differential equation model? Is `malariasimple` a difference equation? How does `malariasimple`'s discrete time step work? Is `malariasimple` a discretization of ICDMM, or in what other way is it related to ICDMM? What is the relationship between ICDMM and `malariasimulation`? What time step should the user consider choosing when running `malariasimple`, and how might this choice affect the model in theory and in practice?

We have clarified that *malariasimple* is formulated using difference equations with state variables updated at fixed time steps.

**Lines** [**80-85**]. “*malariasimple* is a discrete-time compartmental model of malaria transmission that captures the coupled dynamics of human and mosquito populations. The model is formulated using difference equations with state variables updated at fixed time steps, and converges to the equivalent differential equation formulation as the time step length approaches zero. *malariasimple* builds upon the previously developed Imperial College Deterministic Malaria Model (8) (Supporting information S2.1 provides a detailed description of the differences between model versions).“

However, we have chosen to avoid focussing on differences between ICDMM and malariasimple in the main text as this is not necessary to the user’s understanding of the model and could confuse the more relevant comparison with malariasimulation. We do nevertheless agree that more clarity should be made available to the interested reader who may be familiar with ICDMM and have therefore added a paragraph in S2 which provides more details on how *malariasimple* builds upon ICDMM with signposting found in the main text.

S2.1 – Relationship between ICDMM, malariasimple, and malariasimulation

“The Imperial College Deterministic Malaria Model (ICDMM) [mrc-ide.github.io/deterministic-malaria-model] is a continuous-time compartmental model formulated using ordinary differential equations and developed as a simplified approximation to the individual-based model malariasimulation. malariasimple builds upon the conceptual framework of ICDMM but extends it in several key ways. First, it introduces a discrete-time formulation using difference equations allowing greater flexibility in implementation. Second, it includes both deterministic and stochastic variants, enabling users to capture either smooth average dynamics or random variation in transmission. Third, it substantially expands the representation of interventions, which are only treated in a limited way in ICDMM. Finally, the model interface and workflow are designed to closely mirror malariasimulation, allowing users to move seamlessly between the fast compartmental approximation and the more detailed individual-based model."

We have included a paragraph briefly introducing the tuneable parameters (including time steps per day) in the main text.

Lines [139-148]. “*malariasimple* includes a small number of tuneable numerical configuration settings that control the trade-off between runtime and approximation accuracy. These include the number of time steps per day, the number of biting heterogeneity groups, the stratifications of human ages, and the number of sub-compartments used to approximate the latent delay between exposure and blood-stage infection (see vignette *model_introduction.Rmd* for further details). Increasing the number of time steps or compartments increases the numerical resolution of the model and generally accuracy to the individual model, but increases runtime. All results presented in this paper were generated using the default model configuration and should therefore be interpreted as conditional on this.”

In addition, in the latest version of malariasimple we have included a vignette on speed/accuracy trade-offs from tuneable parameters. This details the effect of varying tsd on accuracy to the individual model.

**1.2** Likewise, the explanation of the stochastic version of the model in the main text (line 139 and line 142) is extremely vague.

We agree it should be strengthened and the ‘model structure’ section has been expanded to provide further details about the stochastic model and how it is implemented.

Lines [158-169]. “In the stochastic formulation, transitions between human compartments are modelled as probabilistic processes. For each sub-compartment and time step, the total number of individuals leaving is first sampled from a binomial distribution, with probability determined by the combined transition rates. These individuals are then distributed across the possible destination compartments using a multinomial distribution in proportion to their respective transition rates. This approach ensures that competing risks are handled explicitly and that population counts are conserved. The stochastic formulation therefore introduces demographic variability into the model while retaining the same underlying structure and parameters as the deterministic version. In large populations, the stochastic model converges to the deterministic solution, but in smaller populations or low-transmission settings it captures variability and extinction dynamics that are not represented in the deterministic model.”

**1.3** Supplement S2 and the R Vignettes contain valuable further information about the models. I understand and accept that the main text of this paper does not need to provide a comprehensive mathematical description of every aspect of the model, and that interested readers can refer to these additional resources. However, I strongly believe that, at the very least, a few additional elaborations of what these models are actually doing is needed in the main text.

This has been done. Further details summarising how state transitions are calculated and tuneable parameters have been added in the ‘Model Structure’ section [Lines 139-148], see response 1.1 for more details.

**1.4**`malariasimple` evidently has several key tunable simulation configuration parameters (time steps per day, number of biting compartments, and so forth) which possibly have an important effect on both runtime and accuracy. I noticed that these are discussed nicely in lines 74 to 84 of model_introduction.Rmd on Github (malariasimple/vignettes/), but not in the paper. Again, I don't expect these to be analyzed comprehensively in the main text. But I believe that it ought to be clarified that results presented in the text (e.g., Figure 2) are contingent on certain simulation configuration settings, what choices about these settings were made to obtain the illustrated results, and some basic information or insight about the role of these parameters provided to the reader (without them having to delve into the Rmd files or elsewhere).

Assuming that it is indeed intended for `malariasimple` to have a user-tunable time step, choosing the value of this time step is particularly interesting (and, sometimes, challenging) from the perspective of assessing the accuracy and runtime of simulation or inference for discrete time models. Are runs of `malariasimple` with different time steps best understood as different models altogether, or approximations of some underlying model with differing accuracy? Is the recommended time step for simulation the same as the recommended time step for inference? However, not only is this key configuration choice not discussed in the paper, its value is also somewhat nontrivial for someone to discern even if they are delving into the code itself (as there's a "default_dt" of 1 in the dust.R file, versus a "tsd = 4" (time steps per day) in the get_parameters.R file).

We agree, and as outlined in response 1.1 we have included a paragraph briefly introducing the tunable parameters in the main text. In the ‘model_introduction.rmd’ vignette, we have included information on the default values of the tuneable parameters and how to change them. We have also added a vignette ‘speed_accuracy_parameters.rmd’ detailing the effect of various configuration settings on accuracy to malariasimulation and runtime.

**1.5** To improve the paper, the effect of these various configuration settings on accuracy and runtime could be analyzed and presented in the paper. However I acknowledge that developing comprehensive results in this direction would be a major undertaking, and is not needed for the paper to still be a useful description and explanation of `malariasimple`, provided that some additional explanatory text is added.

Please see response 1.1.

**1.6** There are many prior research efforts to: approximate individual based models with related differential or difference equation models, and/or learn IBM parameters using fast approximations of the underlying model. Do the authors think that it might be helpful to give some indication explaining how their work fits into these efforts? The current draft of the paper is extremely sparse in this respect, even by the standards of a software paper. Even just two or three extra sentences would, in my opinion, substantially improve the paper.

We agree and have reworked the introduction to introduce our approach in the context of the recent popularity of emulators.

Lines [54-63] “One approach that has gained popularity in recent years is the use of emulators to create fast approximations of individual-based transmission models (e.g. (3–7)). These emulators are generally trained on many runs of the original model across the input parameter space and, once trained, can generate outputs almost instantaneously, enabling rapid Bayesian calibration and uncertainty analysis. However, this approach often comes at the cost of a computationally intensive upfront training stage and is fundamentally black-box in nature. This can make it less straightforward for users to explore modified model structures, for example by introducing a novel intervention or incorporating sensitivity to a particular environmental variable, as the emulator may need to be retrained.”

Minor concerns

**1.7** Line 26 and line 39. In the first paragraph, these models are described as fast, but in line 39, the high computational cost is emphasized. I think it's worth clarifying that the models can be fast relative to other forms of investigation, but still slow enough to be inconvenient for other purposes.

We agree and have clarified the models are fast *relative to field studies* in lines 25-29.

“Process-based transmission models help to address this need: relative to field studies, they provide fast, low-cost evaluations of intervention portfolios, allow exploration of strategies in future contexts such as climate change, and enable counterfactuals that would be impractical, unethical, or too slow to test empirically”

Line 75. It says the previous continuous model is "known as" the Imperial College Deterministic Malaria Model (ICDMM). However, the provided reference doesn’t seem to use the term, except once within a URL in its supplementary material. As mentioned in Major Concerns, rather than introducing ICDMM just in this way, it might be more helpful to give a bit of background of what ICDMM actually is and how it relates to malariasimulation.

In addition to response 1.1 the reference for ICDMM has now been changed to something more helpful (the github repo for the package). As in the previous comment, further detail regarding the relationship between malariasimulation, malariasimple, and ICDMM has been provided in the supplementary and sign-posted in the main text.

**1.8** Line 140. "Realisation" doesn’t seem like the best word to describe this. Instead, the "deterministic option" or "deterministic model" or "deterministic version" might be clearer.

Agreed. We have changed to ‘deterministic option’.

**1.9** Line 254. It states that the work was performed using the "Imperial College High Performance Cluster." The Imperial College RCS requests that such usage is described in publications according to the following page, but this does not seem to have been included.

<https://icl-rcs-user-guide.readthedocs.io/en/latest/support/citing-rcs/>

Thank you for pointing out. The appropriate statement has now been added to the acknowledgements.

**1.10** A more general problem here is informing the reader in more detail what sort of computational resources were made available to generate the results in the paper, and what the implications are. For example, dust2, a key dependency of `malariasimple`, advertises its support for "Running systems in parallel on multi-core machines." Does this imply that the fast runtimes reported in the paper for `malariasimple` were only achieved due to massive parallelization enabled by access to a high end cluster? Was `malariasimulation` able to take advantage of parallel processing, or is it unable to benefit from such resources? Is it possible to comment on what runtime performances might be expected by users who are thinking of running the `malariasimple` on more humble computational resources? Is it interesting or important to think about how the models might differ in memory requirements? Is `malariasimple` deployable in low-resource conditions (e.g., someone's basic laptop computer), or does it require higher performance computing?

dust2 supports running systems in parallel and users with access to multi-core machines may choose to use this functionality (e.g. for running multiple stochastic simulations). This support is now noted in lines 91-94 “These parameters are then passed into the main model which is written using odin2 (9), a domain-specific language in R which compiles to C++ for efficient simulation and provides support for parallel processing.” However, this functionality has not been used in the example simulations presented in the paper and hence similar runtimes can be expected from a standard laptop computer. This has now been clarified in the Figure 2 caption “All runs were executed on the Imperial College High Performance Cluster for consistency, however similar runtimes may be expected on a standard laptop computer”

**1.11** S1. The file contains Microsoft Word features (tracked changes), and could consider removing this before submission.

Thank you. We have removed tracked changes from the file.

**1.12** S1 final paragraph. Is "wit" used accidentally instead of "with" anywhere?

Well spotted. Amended.

**1.13** S1.4 To clarify: were MCMC chains initialized with a sample from the prior, or some other method?

Initialised with a sample from the prior. This has now been clarified in the methods (S1.4). . “Each of four chains was initialised with a sample from the prior and run for 5,000 iterations with the first 500 iterations discarded as ‘burn-in’.”

**1.14** S1 does not have references appearing at the end.
Thanks, now added.

**1.15** S2. In the first paragraph of S2.1.2, f_T is defined merely as a "treatment rate" whereas in Table S2.1, f_T is defined as "proportion of cases treated promptly." The second definition makes more sense to me based on the model structure, some more care in defining this parameter precisely may be needed.

Agreed. The second definition is now given in S2.1.2 (now S2.2.2)

1.16 model_introduction.Rmd line 82. "Increasing the number of time steps or compartments will all add to runtime but may reduce accuracy." Is this a typo, would it make more sense if they increased runtime and increased accuracy?

Thanks. Now corrected to ‘add to runtime but may improve accuracy’.

Reviewer #2

This article presents a concise piece of software with a clear and worthwhile objective. The software is likely to be useful for a range of applications, and it's clearly within scope as a software paper in PLOS Computational Biology.

We* reviewed both the paper and the software code, and as per the journal's instructions, we tried to run the reproduce the results in the paper using the software and code provided:
"A prerequisite for publication is that the results described in the paper must be reproducible when peer reviewers or editors choose to run the software on the deposited dataset using the parameters provided." (review invitation email, and here similarly: <https://journals.plos.org/ploscompbiol/s/materials-software-and-code-sharing>)

**2.1** Whilst the authors provided nice clear guidance on how to do this in a standalone GitHub repository, unfortunately it was not possible to rerun the analysis code as many of the functions required had not been uploaded to the repository. We attempted to flag this with the repository owner directly via GitHub (<https://github.com/DebbieShack/malariasimple_paper/issues/1>) and then via email. However, we didn't receive a response before the agreed review deadline so we are unable to complete the review and check this prerequisite. We would be very happy to review a resubmission with this missing functionality included.

We would like to apologies for this. The corresponding author was on maternity leave at the time and missed the email. The GitHub repository has now been amended to include the missing functions and should now be fully reproducible.

We didn't complete a full review of the paper or software in this round since we couldn't run the example code to fully understand it. However we provide below some comments and suggestions we made from our initial review, which could be addressed in a revised version.


Paper:

**2.2** The visual and descriptive comparison between malariasimple and malariasimulation results was clear, but a quantitative discussion of the differences may be useful (that is, building a little more on Figure 3 highlighting the model differences in the text). What can we expect the average model difference to be? Is this acceptable?

Our aim in Figure 3 was to demonstrate that, under a representative seasonal transmission scenario incorporating several important model features (including seasonality, ITN distribution, and SMC), malariasimple closely reproduces malariasimulation. However, in the revised manuscript, we have strengthened this comparison by expanding Figure 3 across a broader range of transmission intensities (Figure S1.2), so that agreement can also be assessed in low-, moderate-, and high-transmission settings.

While we agree that quantitative comparison is valuable, the magnitude of the difference between the models depends on the specific epidemiological setting, intervention profile, and output considered. Because malariasimple introduces several simplifying approximations relative to malariasimulation, the resulting bias is not expected to be constant across scenarios. Similarly, whether a given level of difference is “acceptable” depends on the purpose of the analysis, the reliability of the input parameters and the precision required for the research question being addressed. We have clarified this in the revised text with the statement,

“*To ensure an acceptable level of accuracy for a given scenario and research objective, we recommend that new simulation experiments be benchmarked against equivalent malariasimulation runs to confirm that the simplified model provides an adequate approximation*”

**2.3** The paper says: "To avoid the need for a model initialisation burn-in period, the simulation is initialised at endemic equilibrium using analytical solutions detailed in Griffin 2016 (5)."
But surely this only *lessens* the need for burn in, since the equilibrium won't hold under the external forcing and intervention scenarios used to set up the simulation? Or is there a reason this isn't the case?

The reviewer is correct. The analytical solutions provide endemic equilibrium in the absence of external forcing. To account for the effects of past interventions on disease states and immunity levels, the simulation needs to be run from introduction of interventions. This has been clarified in the main text.

**Lines [171-178]** “To reduce the need for a model initialisation burn-in period, simulations are initialised at endemic equilibrium using analytical solutions detailed in Griffin 2016 (10). These solutions provide equilibrium conditions for a given treatment coverage and baseline entomological inoculation rate (EIR) in the absence of any time-varying external forcing. The resulting stratified infection states and immunity levels are used as initial conditions. When including interventions in the model, simulations are run forward from their time of introduction, allowing the system to evolve from this baseline equilibrium.”

**2.4** The paper says: "Evidence indicates that the protective efficacy of ITNs is distributed across the community though a mass effect, as it is thought mosquitoes feed on people randomly in the community resulting in a minimal difference in malaria prevalence between users and non-users (7,8). Therefore, for both distribution methods the proportion of the population benefiting from an ITN is assumed to be fixed, with time-varying ITN coverage captured by scaling r_ITN and d_ITN according to current users / maximum users."
This is a computationally efficient implementation and therefore a completely reasonable choice in this software. But I think the justification here is a bit too strong, since it implies this isn't an issue. In fact, you demonstrate and discuss later that this assumption leads to a divergence with malariasimulation. Surely if this was as unimportant as claimed here, then this implies malariasimulation is incorrectly parameterised? We suggest toning down the language here to acknowledge that it is a simplification (though not a terrible one).

We agree and thank you for highlighting. We have reworded to “*Therefore, for both distribution methods we make the simplifying assumption that the proportion of the population benefiting from an ITN is fixed…*” to highlight that this is indeed a simplification.

**2.5** We know that artemisinin-combination therapies are a key malaria intervention tool, with their efficacy called into question in light of emerging artemisinin resistance. Therefore, we think there is potential to add treatment coverage as a key intervention of the model (rather than only ITN/SMC). We note there are parameters related to treatment in the code architecture (daily_ft seems most appropriate from a quick review), and Figure 1 shows state transitions (e.g. Diseased to Asymptomatic, Sub-patent to Treated) that would be influenced by treatment coverage rate. It could be worth adding a couple of paragraphs addressing the impact of this parameter, and a vignette exploring its appropriate use.

Explanation regarding how treatment therapies have been included in the model is given in the supplementary materials S2.3.1.

S2.3.1. Defining Treatment Therapies “”

Malaria treatment is represented explicitly in the model through the inclusion of the *Treated* ($T$) and *Prophylactically Protected* ($P$) states. A user-defined proportion of cases, $f_{T}$, is transferred to the *Treated* state, from which individuals then move to the $P$ state before returning to the *Susceptible* ($S$) state (see Figure 1 in the main text). Individuals in both $T$and $P$ are assumed to be protected from further infection. The time spent in each state is assumed to follow an exponential distribution, such that individuals leave the $T$ and $P$ states at constant rates $r_{T}$ and $r_{P}$, respectively.

Individuals in the $T$ state are assumed to remain infected, but to have reduced infectivity to mosquitoes relative to untreated diseased individuals. Their contribution to the human-to-mosquito force of infection ($FOI_{v}$) is represented by the term $c_{T}T[i,j,k]$, where generally $c_{T}<c_{D}$. Here, $c_{T}$and $c_{D}$are compartment-specific per-capita infectiousness parameters that scale the contribution of individuals in $T$ and and *Diseased* $(D)$ states to $FOI_{v}$ respectively.

This explanation is now sign posted in the main text.

**Lines [185-187]** “Full mathematical formulations and parameter definitions for treatment therapies, ITNs and SMC are provided in the Supporting Information S2.3.”

Demonstration of how it can be implemented in practice is now given in the basic_model_run.rmd vignette.

**2.6** Figure 3 – it is hard to see the stochastic and malariasimulation trajectories (granted, due to their substantial overlaps with the deterministic trajectory). Is it possible to make this clearer?
We appreciate the issue and have experimented with several different iterations of this figure for this reason. Nevertheless, the purpose of the figure is to demonstrate the similarity between the outputs of the three models and in trying to separate the trajectories (to make it clearer) the comparison in our opinion becomes less clear. We feel that panel B and D which specifically highlight the differences is the best way of presenting the results in an unbiased manner.

**2.7** L54 typo: "To address, we developed malariasimple ..." -> "To address this need, we developed malariasimple ..."

Thanks, corrected now.

Software:

2.8 The installation instructions were clear and automatically installed dependencies and the software successfully on 2 of 3 machines we tried on. The machine that failed was using a slightly older version of R, and failed on compilation of C++ code when installing the dust2 library, which is a requirement for malariasimple. This is likely due to undocumented incompatible versions of R and C++ for dust2 - which is listed as still being a work in progress package and not necessarily stable. Some version clashes are inevitable, but setting a required minimum R version in the dependencies of either malariasimple or dust2 would help remove this installation friction.

The vignettes looked good, but we noticed some minor issues:
Basic_Model_Run: the last cell (5 stochastic sims at the same time) repeatedly crashed the R session on a laptop, and had to be reduced to only 2 stochastic sims to be able to run. We suggest limiting the computational burden for steps like these in the vignettes.
comparison_with_malariasimulation: malariasimulation was a dependency here, and the vignette wouldn’t run without malariasimulation installed. We suggest linking to docs / installation instructions for malariasimulation
custom_outputs: Typo (line 23), it should say “similar to the method used in malariasimulation” not “malariasimple”
ITN: “Mean ITN efficacy” (red line) doesn’t show in the last plot
model_introduction: the images do not show properly, as the directories are misspecified. Needs to be set to the vignette directory


We would recommend using the goodpractice R package to run some automated checks, as when we ran it it picked up on some things that could have been done better. They are mostly minor, but cover some aspects of code quality (especially the last 4) that will make the software easier and safer for other people to maintain in the future. The goodpractice package and docs will provide more explanation of alternatives and why these things matter:
- Writing some more unit tests
- Adding URL & BugReports to DESCRIPTION
- Consistently using ‘<-‘ for assignment instead of “=”
- Avoiding long code lines
- Avoiding sapply()
- Avoiding 1:length(…) or 1:nrow(…) etc.

*This article was co-reviewed by Yuval Berman (PhD student) and Nick Golding (senior academic) at the University of Western Australia. We discussed the review process, reviewed separately and focussed on different aspects, and then discussed and combined our review notes before preparing the final review report.

Thanks for these helpful suggestions. We have made the changes to the vignettes as suggested and made many improvements as suggested by the goodpractice package.

Reviewer #3

Overall, this is a well-written manuscript describing a new R package (“malariasimple”) that promises to be a useful tool for rapid simulation of malaria control scenarios. The program is based on a previously validated compartmental model (“malariasimulation”), with simplifications that are based on reasonable modeling assumptions. The authors demonstrate substantial improvements in computational efficiency in malariasimple relative to malariasimulation and show that the two models generate similar outputs in at least some scenarios. It would, however, be helpful for the paper to include more detail about the model structure, example use cases, and potential applications of the package (detailed comments below).

We agree, please see response 1.1

Specific comments
**3.1**. Line 76 – It would be helpful (even in supplementary materials) to include further details on the compartmental model structure. Although the basic model has been described elsewhere, there are certain features of the model that may be counterintuitive to a reader (e.g.: explicitly modeling a diseased/untreated compartment) and would require them to go to through malariasimulation papers to understand.

A paragraph explaining how the four infection states differ has been added to section S2.2 in the supplementary.

“The six human compartments are intended to represent epidemiologically distinct infection and treatment states (See Figure 1 in main text). Within these, we explicitly distinguish four infection states: *Diseased* (but untreated), *Treated* (clinical disease that received prompt and effective treatment), *Asymptomatic*, and *Sub-patent*. Each state differs in their onward infectivity to mosquitoes, their probability of being detected by surveillance (and hence their contribution to measured malaria prevalence), and whether they are classified as a clinical ‘case’ when comparing model outputs with malaria case datasets (1).”

**3.2**. Lines 146-7 -How adaptable is the model is to scenarios/interventions other than those described here (for example vaccination). Would users need to wait for those applications to be developed by the authors or can the model structure be adapated to uses beyond those currently implemented?

The hope is that users will adapt to suit their own needs (with caveats). This has been clarified in the conclusion

**Lines [411-421]** “It has been written and documented with the intention of being intuitive and interpretable for users with basic knowledge of infectious disease modelling and R programming, and is designed as a flexible framework that can be adapted to specific research questions and settings. We therefore intend *malariasimple* not only as a tool for the applications presented here, but also as a model structure that users can extend for their own research needs, including by adding new interventions or alternative epidemiological processes. However, users should be aware that altering model structure and parameterisation could generate misleading results as many of the parameters (outlined in (14)) were fit in parallel and are therefore conditional on each other and the underlying structural assumptions in the model.”
**3.3.** Line 160 – “Delta” is the retention rate here, whereas in S2.2.1 (p3) it is defined as “expected retention time.”

Thank you, we have corrected in the main text that delta refers to a retention period rather than a rate.

**3.4.** Line 175 – I’m not entirely sure what “mass effect” means here (community effect?). I think it may be a bit strong to say that mosquitoes feed randomly and with “minimal difference in malaria prevalence between [net] users and non-users”; citation 7 shows that there is a community effect of ITN use but doesn’t compare community and individual effects, citation 8 suggests that the size of the individual effect varies with malaria prevalence. From a modeling perspective, it is likely fair to ignore heterogeneity in usage in this case (i.e. assume average usage) without going so far as to state that ITNs work regardless of whether they’re used.

We have replaced the term ‘*mass effect’* for *‘community effect*’. We have also toned down the language by describing a ‘*minor* difference in malaria prevalence’ and clarified that we are making a simplifying assumption here

**Lines [208-215]** “Evidence indicates that the protective efficacy of ITNs is distributed across the population though a community effect, as it is thought mosquitoes feed on people randomly in the community resulting in only minor differences in malaria prevalence between users and non-users (12,13). Therefore, for both distribution methods we make the simplifying assumption that the proportion of the population benefiting from an ITN is fixed, with time-varying ITN coverage captured by scaling $r_{ITN}$ and $d_{ITN}$ according to current users / maximum users.”

**3.5**. Lines 257; 307-309 – For the scenarios depicted in Figs. 3 and 4, it would be helpful to have more specifics: what data did the authors fit the model to? Is this only the prevalence data (shown in Fig. 3) or was additional (incidence) data used? If this is synthetic data some details on how it was generated would be useful. Was the data aggregated? stratified by covariates?

The simulations are not fit to real data but an arbitrarily defined number of people positive for malaria in a given number of people tested. The credible intervals represent propagated uncertainty due to sampling input parameters from posterior distributions previously derived by fitting malariasimulation to data. This is explained in the Methods section given in the supplementary materials (S1), but for clarity we have added a very brief summary of the method in the results section of the main text.

**Lines [290-293]** “We compared both the deterministic and stochastic versions of *malariasimple* with *malariasimulation* under an identical illustrative seasonal-transmission scenario, while propagating parameter uncertainty through repeated simulations using parameter sets sampled from previously estimated posterior distributions (14).”

**3.6.** Because there is reason to think that stochastic and deterministic models are more likely to differ from each other in very low prevalence settings (as the authors acknowledge), it might make sense to provide the outputs shown in Fig. 3 over a range of prevalences or transmission intensities (as is done in Fig. 4).

We have included an expanded version of Figure 3 in the supplementary (Figure S1.2 which includes low and high transmission scenarios in the supplementary materials.

**3.7**. Lines 278-280 – I wonder if predictions based on this approximation might deviate more from malariasimulation under extremes of host heterogeneity (i.e. in relative biting rates) – as in the last comment, it might be interesting to compare malariasimple and malariasimulation over a range of heterogeneities.

In the latest version of malariasimple we have included a vignette on speed/accuracy trade-offs from tuneable parameters (including number of biting groups). This demonstrates the effect of increasing stratification of heterogeneity groups on similarity to malariasimulation.

**3.8**. “The model captures both the mean dynamics and the uncertainty
around them.” This seems strong to me considering that a single simulation (with unknown parameters) is shown.

We agree this is too strong. Details on population and transmission parameters have now been given in the methods section, with direction to the R code for full parameterisation. The sentence in the main text has been adjusted to state “…demonstrating that, *in this example*, the model effectively captures both the mean dynamics and the uncertainty around them”.
