# Supplementary material for: malariasimple: An R package for fast simulations of malaria transmission
Source: PLoS Comput Biol. 2026 Sep 1;22(9):e1013687. doi: 10.1371/journal.pcbi.1013687 (PMC13561361; doi:10.1371/journal.pcbi.1013687)
Supplement: Supplementary file 4 [file pcbi.1013687.s004.docx]

**Reviewers' comments:**

Reviewer's Responses to Questions

**Comments to the Authors:
Please note here if the review is uploaded as an attachment.**

Reviewer #1: Various helpful code improvements are described in the Response to Reviewers, but it looks like these changes are still on a feature branch on the software repository. It would be good to get them merged to the main branch, or else indicate to readers where to go to obtain them.

We thank the reviewer again for their helpful comments in the previous round, which led to several useful improvements to the code and documentation. The updates were initially available on the working branch, but they have now been merged into the main branch. The merged version also includes several additional minor improvements made in response to recommendations from Reviewer #2.

Line 192. “The stochastic model converges to the deterministic solution,” I wasn’t completely certain on the usage of the word “converge” given its more rigorous mathematical usages (<https://en.wikipedia.org/wiki/Convergence_of_random_variables>); I might have rewritten it “In large populations, the stochastic model more closely approximates the deterministic solution…” However, I am happy to be corrected if the authors have a reason for choosing the word “converge.”

Agreed that ‘converges’ is not the most appropriate word in this context. We have amended now to “*In large populations (for example, over 100,000), stochastic simulations begin to closely approximate the deterministic solution, whereas in smaller populations or low-transmission settings they capture variability and extinction dynamics that are not represented in the deterministic model.”*

Reviewer #2: We* commend the authors again on this novel work and their thoughtful and considered responses to all reviewers’ comments. We believe these changes have made the paper and package stronger. Our comments here are minor and we leave them at the author’s discretion. We do not feel the need to review the paper again after these have been rectified.

Code

We thank the authors for updating the changes we proposed and for making use of the goodpractice package. Most importantly, we note that the Git repository seemed to be updated under a new branch (working), and this should be merged across to main ahead of publication.

Many thanks for your many helpful comments which have certainly improved the model code and documentation. The updates made in the previous round of revisions, plus updates addressing the further comments below have now been merged to main.

The vignettes are a great tool for users to become more familiar with malariasimple, and we commend the authors on the great work. Some minor notes include:

- custom_outputs has the PfPR1-5 of SMC recipients going down much earlier than the SMC intervention is specified to have taken place. Is this a plotting bug?

SMC distribution type has now been set to “correlated” in the vignette to produce a more expected output.
- We still think, as reviewer #3 raised, that it is worth thinking about how transferable the package is to other interventions. For example, int_group is an argument specifically set to one of four options: either ITN only, SMC only, both, or none. If we were to create a vaccine intervention, does this break the get_custom_output function? It also is not immediately clear why PfPR1-5 is reported but the smc_ages variable is defined as 1,2,3 year olds.

Agreed that the current encoding of interventions limits the ease with which new intervention types can be added. In the present version of malariasimple, int_group refers specifically to the four intervention combinations currently supported: no intervention, ITN only, SMC only, or both ITN and SMC. Adding another intervention, such as vaccination, would therefore require extending the intervention structure and updating the relevant output-processing functions. We have clarified this limitation in the documentation for get_custom_output(). In a future iteration of malariasimple, we plan to revise the way interventions are encoded so that additional intervention types can be introduced more easily.

We have also added an explanation to the get_custom vignette clarifying why selecting the age categories with lower bounds of 1, 2, and 3 corresponds to PfPR among children aged 1–5 years for the specified age_vector.
- In basic_model_run vignette, the units of clinical incidence should be specified. Are these cases per individual, or per 10k? The plots in section 2 and 3 differ by orders of magnitude, even though they are reportedly showing the same measure - some clarity here would be useful.

Thanks for pointing out this inconsistency. Clinical incidence is now reported consistently as a per-capita rate, with units of cases per person per day stated explicitly on all relevant plot panels.
- ITN - the “Mean ITN efficacy” (red line) is still not showing in the last plot.

Well observed. The plot now draws this series alongside total ITN usage, with a short explanation of what it represents.

The goodpractice package still makes a few recommendations regarding code improvement. However, at this stage most are minor and we leave it at the author’s discretion as to whether to implement the last few recommendations:
- Writing unit tests for all functions
- Adding URL field and BugReports field to DESCRIPTION
- Using ‘←’ for assignment instead of ‘=’ (there are still two instances where this has not been rectified, inst/odin/malariasimple_deterministic.R:399 and inst/odin/malariasimple_stochastic:486)
- Avoiding long code lines
- Avoid sapply() (in the Basic_Model_Run) vignette

Thanks for pointing out these remaining areas for improvement. We have now applied the reviewers recommendations

We note it is required by the journal to be able to reproduce all figures in the paper, however understandably the figures take too long to produce on a normal laptop since they perform extensive computations and parameter sweeps, so we have not reproduced them (with the exception of Figure 3, which was successfully reproduced). However, one minor comment is that in the script Shackleton26_simulations.R, line 31 needs to be updated (remove the _revised).

Thanks. Typo on line 31 is now fixed.

Paper

The paper is well written and introduces the software package well. We note some minor corrections:

- We recommend being explicit with what a ‘large population size’ means in line 192 (10 thousand? 100 thousand?), when comparing similarity between deterministic and stochastic outputs.

Thanks for the comment. We have clarified in the text that by ‘large’ we mean 100,000+. “*In large populations (for example, over 100,000), stochastic simulations begin to closely approximate the deterministic solution, whereas in smaller populations or low-transmission settings they capture variability and extinction dynamics that are not represented in the deterministic model.”*
- The claim that mosquito biting rates increase with body surface area (line 145-6) would benefit from a citation. For example, this is the one commonly cited in OpenMalaria reference documentation when making the same claims: Port, G., Boreham, P., and Bryan, J. H. (1980). The Relationship of Host Size to Feeding by Mosquitoes of the Anopheles Gambiae Giles Complex (Diptera: Culicidae). Bulletin of Entomological Research 70, 133–144

Thanks for the suggested citation. Agreed this is appropriate here. It has been added to main text.
- In the supplementary material S2.4, the authors say “We then allocate those individuals to each destination in proportion to their respective rates, ensuring that the total number leaving never exceeds the number present at the start of the time step” - however this does not seem to match the maths provided. Furthermore, this would be the wrong way to implement stochasticity, as instead the flow into each individual compartment should be a binomial realisation, not a proportion. The correct way does seem to be reflected in the maths and code provided, so we recommend updating the word description to also reflect this.

Thanks for noticing this error. We have corrected by replacement with the following paragraph: *“To simulate competing risks between different compartments, we first draw the total number of individuals leaving a sub-compartment from a Binomial distribution, with the probability determined by the sum of outflow rates over the time step. The destination of each individual is then sampled from a Multinomial distribution, with destination probabilities proportional to the corresponding transition rates. This ensures that the total number leaving a sub-compartment cannot exceed the number present at the start of the time step. In our implementation, this Multinomial allocation is represented using an equivalent sequence of conditional Binomial draws, as Multinomial draws are not directly supported in odin.dust. The indices i, j, and k refer to positions in the three-dimensional state arrays, corresponding to age group, biting heterogeneity group, and intervention category, respectively.”*

- Typo, line 121-2: “late instar lave → larvae”

Thanks, corrected.
- Line 170 is poorly worded, would benefit from being more specific: “accuracy compared to the individual-based malariasimulation model, but increases runtime” (or similar).

Thanks, amended as suggested. Sentence now reads: *“Increasing the number of time steps or compartments increases the numerical resolution of the model and accuracy compared to the individual-based malariasimulation model, but increases runtime.”*
- Typo, line 221: “assumed to wane”

Thanks. Corrected.
- Line 220: for completeness, it may be useful to provide the parameter for a mosquito that survives and successfully bites?

We have now clarified this as $S_{ITN}$
- SI 1: Figure S1.2 is floating - it has no reference in the text.

We thank the reviewer for raising this point. Figure S1.2 is referenced in the main text and has a detailed legend describing the comparison between malariasimple and malariasimulation across low, moderate, and high transmission settings. We therefore consider the figure to be adequately introduced and explained.
- SI 2: S2.2.4 typo: “clinincal recovery”

Thanks, corrected.
- SI 2: equation bottom of page 3 - we assume the integral should be with respect to time (i.e. add dt at the end).
Thanks, corrected.
*Nick Golding (Professor) and Yuval Berman (PhD student), University of Western Australia
