# Supplementary material for: malariasimple: An R package for fast simulations of malaria transmission
Source: PLoS Comput Biol. 2026 Sep 1;22(9):e1013687. doi: 10.1371/journal.pcbi.1013687 (PMC13561361; doi:10.1371/journal.pcbi.1013687)
Supplement: Peer review history

# Peer review: Decision Letter 0

**DOI:** 10.1371/journal.pcbi.1013687.r001

**Authors:** Roger Dimitri Kouyos, Oliver Eales

19 Jan 2026

PCOMPBIOL-D-25-02244

malariasimple: An R package for fast simulations of malaria transmission

PLOS Computational Biology

Dear Dr. Shackleton,

Thank you for submitting your manuscript to PLOS Computational Biology. After careful consideration, we feel that it has merit but does not fully meet PLOS Computational Biology's publication criteria as it currently stands. Therefore, we invite you to submit a revised version of the manuscript that addresses the points raised during the review process. The reviewers were very positive about the potential contributions of the malariasimple software package. However, there were some problems with the reproducibility of results raised by Reviewers 2 that would need to be fixed before the manuscript could be accepted. Additionally, the work could be improved by more thoroughly explaining and contextualizing the work; there are many specific suggestions from the reviewers that should be carefully considered.

Please submit your revised manuscript by Mar 21 2026 11:59PM. If you will need more time than this to complete your revisions, please reply to this message or contact the journal office at ploscompbiol@plos.org. When you're ready to submit your revision, log on to https://www.editorialmanager.com/pcompbiol/ and select the 'Submissions Needing Revision' folder to locate your manuscript file.

Please include the following items when submitting your revised manuscript:

* A letter that responds to each point raised by the editor and reviewer(s). You should upload this letter as a separate file labeled 'Response to Reviewers'. This file does not need to include responses to formatting updates and technical items listed in the 'Journal Requirements' section below.

* A marked-up copy of your manuscript that highlights changes made to the original version. You should upload this as a separate file labeled 'Revised Manuscript with Track Changes'.

* An unmarked version of your revised paper without tracked changes. You should upload this as a separate file labeled 'Manuscript'.

If you would like to make changes to your financial disclosure, competing interests statement, or data availability statement, please make these updates within the submission form at the time of resubmission. Guidelines for resubmitting your figure files are available below the reviewer comments at the end of this letter

We look forward to receiving your revised manuscript.

Kind regards,

Oliver Eales

Academic Editor

PLOS Computational Biology

Roger Kouyos

Section Editor

PLOS Computational Biology

**Journal Requirements:**

1) Please provide an Author Summary. This should appear in your manuscript between the Abstract (if applicable) and the Introduction, and should be 150-200 words long. The aim should be to make your findings accessible to a wide audience that includes both scientists and non-scientists. Sample summaries can be found on our website under Submission Guidelines:

https://journals.plos.org/ploscompbiol/s/submission-guidelines#loc-parts-of-a-submission

2) Please upload all main figures as separate Figure files in .tif or .eps format. For more information about how to convert and format your figure files please see our guidelines:

https://journals.plos.org/ploscompbiol/s/figures

**Reviewers' comments:**

Reviewer's Responses to Questions

**Comments to the Authors:**

**Please note here if the review is uploaded as an attachment.**

Reviewer #1: Summary

The authors developed the `malariasimple` R package, which implements a simplified, faster version of a malaria transmission model. The structure, model compartments, and modelled interventions are briefly described. The outputs of `malariasimple` are compared to those of the more complex `malariasimulation` individual-based model, showing that `malariasimple` is much faster to run while achieving similar outputs.

I believe that improving the runtime of complex models to enable parameter inference and other analyses is important, and the present work makes a valuable contribution to support such efforts.

Major concerns

The text provides two key pieces of overview information about `malariasimple`: it is a "discrete-time compartmental model," and it is "an update to a previously developed continuous-time compartmental model known as the Imperial College Deterministic Malaria Model (ICDMM)." Although this information is helpful, further clarity and more information is needed.

Although a reference (3) is provided for ICDMM, that reference is also sparse on details. I would appreciate it if the main text briefly provided basic information such as:

Is ICDMM a differential equation model? Is `malariasimple` a difference equation? How does `malariasimple`'s discrete time step work? Is `malariasimple` a discretization of ICDMM, or in what other way is it related to ICDMM? What is the relationship between ICDMM and `malariasimulation`? What time step should the user consider choosing when running `malariasimple`, and how might this choice affect the model in theory and in practice?

Likewise, the explanation of the stochastic version of the model in the main text (line 139 and line 142) is extremely vague.

Supplement S2 and the R Vignettes contain valuable further information about the models. I understand and accept that the main text of this paper does not need to provide a comprehensive mathematical description of every aspect of the model, and that interested readers can refer to these additional resources. However, I strongly believe that, at the very least, a few additional elaborations of what these models are actually doing is needed in the main text.

`malariasimple` evidently has several key tunable simulation configuration parameters (time steps per day, number of biting compartments, and so forth) which possibly have an important effect on both runtime and accuracy. I noticed that these are discussed nicely in lines 74 to 84 of model_introduction.Rmd on Github (malariasimple/vignettes/), but not in the paper. Again, I don't expect these to be analyzed comprehensively in the main text. But I believe that it ought to be clarified that results presented in the text (e.g., Figure 2) are contingent on certain simulation configuration settings, what choices about these settings were made to obtain the illustrated results, and some basic information or insight about the role of these parameters provided to the reader (without them having to delve into the Rmd files or elsewhere).

Assuming that it is indeed intended for `malariasimple` to have a user-tunable time step, choosing the value of this time step is particularly interesting (and, sometimes, challenging) from the perspective of assessing the accuracy and runtime of simulation or inference for discrete time models. Are runs of `malariasimple` with different time steps best understood as different models altogether, or approximations of some underlying model with differing accuracy? Is the recommended time step for simulation the same as the recommended time step for inference? However, not only is this key configuration choice not discussed in the paper, its value is also somewhat nontrivial for someone to discern even if they are delving into the code itself (as there's a "default_dt" of 1 in the dust.R file, versus a "tsd = 4" (time steps per day) in the get_parameters.R file).

To improve the paper, the effect of these various configuration settings on accuracy and runtime could be analyzed and presented in the paper. However I acknowledge that developing comprehensive results in this direction would be a major undertaking, and is not needed for the paper to still be a useful description and explanation of `malariasimple`, provided that some additional explanatory text is added.

There are many prior research efforts to: approximate individual based models with related differential or difference equation models, and/or learn IBM parameters using fast approximations of the underlying model. Do the authors think that it might be helpful to give some indication explaining how their work fits into these efforts? The current draft of the paper is extremely sparse in this respect, even by the standards of a software paper. Even just two or three extra sentences would, in my opinion, substantially improve the paper.

Minor concerns

Line 26 and line 39. In the first paragraph, these models are described as fast, but in line 39, the high computational cost is emphasized. I think it's worth clarifying that the models can be fast relative to other forms of investigation, but still slow enough to be inconvenient for other purposes.

Line 75. It says the previous continuous model is "known as" the Imperial College Deterministic Malaria Model (ICDMM). However, the provided reference doesn’t seem to use the term, except once within a URL in its supplementary material. As mentioned in Major Concerns, rather than introducing ICDMM just in this way, it might be more helpful to give a bit of background of what ICDMM actually is and how it relates to malariasimulation.

Line 140. "Realisation" doesn’t seem like the best word to describe this. Instead, the "deterministic option" or "deterministic model" or "deterministic version" might be clearer.

Line 254. It states that the work was performed using the "Imperial College High Performance Cluster." The Imperial College RCS requests that such usage is described in publications according to the following page, but this does not seem to have been included.

https://icl-rcs-user-guide.readthedocs.io/en/latest/support/citing-rcs/

A more general problem here is informing the reader in more detail what sort of computational resources were made available to generate the results in the paper, and what the implications are. For example, dust2, a key dependency of `malariasimple`, advertises its support for "Running systems in parallel on multi-core machines." Does this imply that the fast runtimes reported in the paper for `malariasimple` were only achieved due to massive parallelization enabled by access to a high end cluster? Was `malariasimulation` able to take advantage of parallel processing, or is it unable to benefit from such resources? Is it possible to comment on what runtime performances might be expected by users who are thinking of running the `malariasimple` on more humble computational resources? Is it interesting or important to think about how the models might differ in memory requirements? Is `malariasimple` deployable in low-resource conditions (e.g., someone's basic laptop computer), or does it require higher performance computing?

S1. The file contains Microsoft Word features (tracked changes), and could consider removing this before submission.

S1 final paragraph. Is "wit" used accidentally instead of "with" anywhere?

S1.4 To clarify: were MCMC chains initialized with a sample from the prior, or some other method?

S1 does not have references appearing at the end.

S2. In the first paragraph of S2.1.2, f_T is defined merely as a "treatment rate" whereas in Table S2.1, f_T is defined as "proportion of cases treated promptly." The second definition makes more sense to me based on the model structure, some more care in defining this parameter precisely may be needed.

model_introduction.Rmd line 82. "Increasing the number of time steps or compartments will all add to runtime but may reduce accuracy." Is this a typo, would it make more sense if they increased runtime and increased accuracy?

Reviewer #2: This article presents a concise piece of software with a clear and worthwhile objective. The software is likely to be useful for a range of applications, and it's clearly within scope as a software paper in PLOS Computational Biology.

We* reviewed both the paper and the software code, and as per the journal's instructions, we tried to run the reproduce the results in the paper using the software and code provided:

"A prerequisite for publication is that the results described in the paper must be reproducible when peer reviewers or editors choose to run the software on the deposited dataset using the parameters provided." (review invitation email, and here similarly: https://journals.plos.org/ploscompbiol/s/materials-software-and-code-sharing)

Whilst the authors provided nice clear guidance on how to do this in a standalone GitHub repository, unfortunately it was not possible to rerun the analysis code as many of the functions required had not been uploaded to the repository. We attempted to flag this with the repository owner directly via GitHub (https://github.com/DebbieShack/malariasimple_paper/issues/1) and then via email. However, we didn't receive a response before the agreed review deadline so we are unable to complete the review and check this prerequisite. We would be very happy to review a resubmission with this missing functionality included.

We didn't complete a full review of the paper or software in this round since we couldn't run the example code to fully understand it. However we provide below some comments and suggestions we made from our initial review, which could be addressed in a revised version.

Paper:

The visual and descriptive comparison between malariasimple and malariasimulation results was clear, but a quantitative discussion of the differences may be useful (that is, building a little more on Figure 3 highlighting the model differences in the text). What can we expect the average model difference to be? Is this acceptable?

The paper says: "To avoid the need for a model initialisation burn-in period, the simulation is initialised at endemic equilibrium using analytical solutions detailed in Griffin 2016 (5)."

But surely this only *lessens* the need for burn in, since the equilibrium won't hold under the external forcing and intervention scenarios used to set up the simulation? Or is there a reason this isn't the case?

The paper says: "Evidence indicates that the protective efficacy of ITNs is distributed across the community though a mass effect, as it is thought mosquitoes feed on people randomly in the community resulting in a minimal difference in malaria prevalence between users and non-users (7,8). Therefore, for both distribution methods the proportion of the population benefiting from an ITN is assumed to be fixed, with time-varying ITN coverage captured by scaling r_ITN and d_ITN according to current users / maximum users."

This is a computationally efficient implementation and therefore a completely reasonable choice in this software. But I think the justification here is a bit too strong, since it implies this isn't an issue. In fact, you demonstrate and discuss later that this assumption leads to a divergence with malariasimulation. Surely if this was as unimportant as claimed here, then this implies malariasimulation is incorrectly parameterised? We suggest toning down the language here to acknowledge that it is a simplification (though not a terrible one).

We know that artemisinin-combination therapies are a key malaria intervention tool, with their efficacy called into question in light of emerging artemisinin resistance. Therefore, we think there is potential to add treatment coverage as a key intervention of the model (rather than only ITN/SMC). We note there are parameters related to treatment in the code architecture (daily_ft seems most appropriate from a quick review), and Figure 1 shows state transitions (e.g. Diseased to Asymptomatic, Sub-patent to Treated) that would be influenced by treatment coverage rate. It could be worth adding a couple of paragraphs addressing the impact of this parameter, and a vignette exploring its appropriate use.

Figure 3 – it is hard to see the stochastic and malariasimulation trajectories (granted, due to their substantial overlaps with the deterministic trajectory). Is it possible to make this clearer?

L54 typo: "To address, we developed malariasimple ..." -> "To address this need, we developed malariasimple ..."

Software:

The installation instructions were clear and automatically installed dependencies and the software successfully on 2 of 3 machines we tried on. The machine that failed was using a slightly older version of R, and failed on compilation of C++ code when installing the dust2 library, which is a requirement for malariasimple. This is likely due to undocumented incompatible versions of R and C++ for dust2 - which is listed as still being a work in progress package and not necessarily stable. Some version clashes are inevitable, but setting a required minimum R version in the dependencies of either malariasimple or dust2 would help remove this installation friction.

The vignettes looked good, but we noticed some minor issues:

Basic_Model_Run: the last cell (5 stochastic sims at the same time) repeatedly crashed the R session on a laptop, and had to be reduced to only 2 stochastic sims to be able to run. We suggest limiting the computational burden for steps like these in the vignettes.

comparison_with_malariasimulation: malariasimulation was a dependency here, and the vignette wouldn’t run without malariasimulation installed. We suggest linking to docs / installation instructions for malariasimulation

custom_outputs: Typo (line 23), it should say “similar to the method used in malariasimulation” not “malariasimple”

ITN: “Mean ITN efficacy” (red line) doesn’t show in the last plot

model_introduction: the images do not show properly, as the directories are misspecified. Needs to be set to the vignette directory

We would recommend using the goodpractice R package to run some automated checks, as when we ran it it picked up on some things that could have been done better. They are mostly minor, but cover some aspects of code quality (especially the last 4) that will make the software easier and safer for other people to maintain in the future. The goodpractice package and docs will provide more explanation of alternatives and why these things matter:

- Writing some more unit tests

- Adding URL & BugReports to DESCRIPTION

- Consistently using ‘<-‘ for assignment instead of “=”

- Avoiding long code lines

- Avoiding sapply()

- Avoiding 1:length(…) or 1:nrow(…) etc.

*This article was co-reviewed by Yuval Berman (PhD student) and Nick Golding (senior academic) at the University of Western Australia. We discussed the review process, reviewed separately and focussed on different aspects, and then discussed and combined our review notes before preparing the final review report.

Reviewer #3: Overall, this is a well-written manuscript describing a new R package (“malariasimple”) that promises to be a useful tool for rapid simulation of malaria control scenarios. The program is based on a previously validated compartmental model (“malariasimulation”), with simplifications that are based on reasonable modeling assumptions. The authors demonstrate substantial improvements in computational efficiency in malariasimple relative to malariasimulation and show that the two models generate similar outputs in at least some scenarios. It would, however, be helpful for the paper to include more detail about the model structure, example use cases, and potential applications of the package (detailed comments below).

Specific comments

1. Line 76 – It would be helpful (even in supplementary materials) to include further details on the compartmental model structure. Although the basic model has been described elsewhere, there are certain features of the model that may be counterintuitive to a reader (e.g.: explicitly modeling a diseased/untreated compartment) and would require them to go to through malariasimulation papers to understand.

2. Lines 146-7 -How adaptable is the model is to scenarios/interventions other than those described here (for example vaccination). Would users need to wait for those applications to be developed by the authors or can the model structure be adapated to uses beyond those currently implemented?

3. Line 160 – “Delta” is the retention rate here, whereas in S2.2.1 (p3) it is defined as “expected retention time.”

4. Line 175 – I’m not entirely sure what “mass effect” means here (community effect?). I think it may be a bit strong to say that mosquitoes feed randomly and with “minimal difference in malaria prevalence between [net] users and non-users”; citation 7 shows that there is a community effect of ITN use but doesn’t compare community and individual effects, citation 8 suggests that the size of the individual effect varies with malaria prevalence. From a modeling perspective, it is likely fair to ignore heterogeneity in usage in this case (i.e. assume average usage) without going so far as to state that ITNs work regardless of whether they’re used.

5. Lines 257; 307-309 – For the scenarios depicted in Figs. 3 and 4, it would be helpful to have more specifics: what data did the authors fit the model to? Is this only the prevalence data (shown in Fig. 3) or was additional (incidence) data used? If this is synthetic data some details on how it was generated would be useful. Was the data aggregated? stratified by covariates?

6. Because there is reason to think that stochastic and deterministic models are more likely to differ from each other in very low prevalence settings (as the authors acknowledge), it might make sense to provide the outputs shown in Fig. 3 over a range of prevalences or transmission intensities (as is done in Fig. 4).

7. Lines 278-280 – I wonder if predictions based on this approximation might deviate more from malariasimulation under extremes of host heterogeneity (i.e. in relative biting rates) – as in the last comment, it might be interesting to compare malariasimple and malariasimulation over a range of heterogeneities.

8. “The model captures both the mean dynamics and the uncertainty

around them.” This seems strong to me considering that a single simulation (with unknown parameters) is shown.

**********

**Have the authors made all data and (if applicable) computational code underlying the findings in their manuscript fully available?**

The PLOS Data policy requires authors to make all data and code underlying the findings described in their manuscript fully available without restriction, with rare exception (please refer to the Data Availability Statement in the manuscript PDF file). The data and code should be provided as part of the manuscript or its supporting information, or deposited to a public repository. For example, in addition to summary statistics, the data points behind means, medians and variance measures should be available. If there are restrictions on publicly sharing data or code —e.g. participant privacy or use of data from a third party—those must be specified.

Reviewer #1: Yes

Reviewer #2: **No:**  as per response to authors, it was not possible to rerun the analysis code as many of the functions required had not been uploaded to the repository

Reviewer #3: Yes

**********

PLOS authors have the option to publish the peer review history of their article (what does this mean?). If published, this will include your full peer review and any attached files.

If you choose “no”, your identity will remain anonymous but your review may still be made public.

**Do you want your identity to be public for this peer review?**  For information about this choice, including consent withdrawal, please see our Privacy Policy.

Reviewer #1: No

Reviewer #2: **Yes:**  Nick Golding and Yuval Berman

Reviewer #3: No

[NOTE: If reviewer comments were submitted as an attachment file, they will be attached to this email and accessible via the submission site. Please log into your account, locate the manuscript record, and check for the action link "View Attachments". If this link does not appear, there are no attachment files.]

**Figure resubmission:**

**Reproducibility:**

?>

# Author comment: Author response to Decision Letter 1

**DOI:** 10.1371/journal.pcbi.1013687.r002

7 May 2026

Attachment Submitted filename: reviewer responses final.docx

# Peer review: Decision Letter 1

**DOI:** 10.1371/journal.pcbi.1013687.r003

**Authors:** Roger Dimitri Kouyos, Oliver Eales

10 Jun 2026

PCOMPBIOL-D-25-02244R1

malariasimple: An R package for fast simulations of malaria transmission

PLOS Computational Biology

Dear Dr. Shackleton,

Thank you for submitting your manuscript to PLOS Computational Biology. After careful consideration, we feel that it has merit but does not fully meet PLOS Computational Biology's publication criteria as it currently stands. Therefore, we invite you to submit a revised version of the manuscript that addresses the points raised during the review process.

Please submit your revised manuscript by Aug 10 2026 11:59PM. If you will need more time than this to complete your revisions, please reply to this message or contact the journal office at ploscompbiol@plos.org. When you're ready to submit your revision, log on to https://www.editorialmanager.com/pcompbiol/ and select the 'Submissions Needing Revision' folder to locate your manuscript file.

Please include the following items when submitting your revised manuscript:

* A letter that responds to each point raised by the editor and reviewer(s). You should upload this letter as a separate file labeled 'Response to Reviewers'. This file does not need to include responses to formatting updates and technical items listed in the 'Journal Requirements' section below.

* A marked-up copy of your manuscript that highlights changes made to the original version. You should upload this as a separate file labeled 'Revised Manuscript with Track Changes'.

* An unmarked version of your revised paper without tracked changes. You should upload this as a separate file labeled 'Manuscript'.

If you would like to make changes to your financial disclosure, competing interests statement, or data availability statement, please make these updates within the submission form at the time of resubmission. Guidelines for resubmitting your figure files are available below the reviewer comments at the end of this letter.

As the corresponding author, your ORCID iD is verified in the submission system and will appear in the published article. PLOS supports the use of ORCID, and we encourage all coauthors to register for an ORCID iD and use it as well. Please encourage your coauthors to verify their ORCID iD within the submission system before final acceptance, as unverified ORCID iDs will not appear in the published article. Only the individual author can complete the verification step; PLOS staff cannot verify ORCID iDs on behalf of authors.

We look forward to receiving your revised manuscript.

Kind regards,

Oliver Eales

Academic Editor

PLOS Computational Biology

Roger Kouyos

Section Editor

PLOS Computational Biology

**Additional Editor Comments (if provided):**

**Journal Requirements:**

If the reviewer comments include a recommendation to cite specific previously published works, please review and evaluate these publications to determine whether they are relevant and should be cited. There is no requirement to cite these works unless the editor has indicated otherwise.

1) Please amend your detailed Financial Disclosure statement. This is published with the article. It must therefore be completed in full sentences and contain the exact wording you wish to be published.

i) State the initials, alongside each funding source, of each author to receive each grant. For example: "This work was supported by the National Institutes of Health (####### to AM; ###### to CJ) and the National Science Foundation (###### to AM).".

If you did not receive any funding for this study, please simply state: u201cThe authors received no specific funding for this work.u201d

**Reviewers' comments:**

Reviewer's Responses to Questions

**Comments to the Authors:**

**Please note here if the review is uploaded as an attachment.**

Reviewer #1: Various helpful code improvements are described in the Response to Reviewers, but it looks like these changes are still on a feature branch on the software repository. It would be good to get them merged to the main branch, or else indicate to readers where to go to obtain them.

Line 192. “The stochastic model converges to the deterministic solution,” I wasn’t completely certain on the usage of the word “converge” given its more rigorous mathematical usages (https://en.wikipedia.org/wiki/Convergence_of_random_variables); I might have rewritten it “In large populations, the stochastic model more closely approximates the deterministic solution…” However, I am happy to be corrected if the authors have a reason for choosing the word “converge.”

Reviewer #2: We* commend the authors again on this novel work and their thoughtful and considered responses to all reviewers’ comments. We believe these changes have made the paper and package stronger. Our comments here are minor and we leave them at the author’s discretion. We do not feel the need to review the paper again after these have been rectified.

Code

We thank the authors for updating the changes we proposed and for making use of the goodpractice package. Most importantly, we note that the Git repository seemed to be updated under a new branch (working), and this should be merged across to main ahead of publication.

The vignettes are a great tool for users to become more familiar with malariasimple, and we commend the authors on the great work. Some minor notes include:

- custom_outputs has the PfPR1-5 of SMC recipients going down much earlier than the SMC intervention is specified to have taken place. Is this a plotting bug?

- We still think, as reviewer #3 raised, that it is worth thinking about how transferable the package is to other interventions. For example, int_group is an argument specifically set to one of four options: either ITN only, SMC only, both, or none. If we were to create a vaccine intervention, does this break the get_custom_output function? It also is not immediately clear why PfPR1-5 is reported but the smc_ages variable is defined as 1,2,3 year olds.

- In basic_model_run vignette, the units of clinical incidence should be specified. Are these cases per individual, or per 10k? The plots in section 2 and 3 differ by orders of magnitude, even though they are reportedly showing the same measure - some clarity here would be useful.

- ITN - the “Mean ITN efficacy” (red line) is still not showing in the last plot.

The goodpractice package still makes a few recommendations regarding code improvement. However, at this stage most are minor and we leave it at the author’s discretion as to whether to implement the last few recommendations:

- Writing unit tests for all functions

- Adding URL field and BugReports field to DESCRIPTION

- Using ‘←’ for assignment instead of ‘=’ (there are still two instances where this has not been rectified, inst/odin/malariasimple_deterministic.R:399 and inst/odin/malariasimple_stochastic:486)

- Avoiding long code lines

- Avoid sapply() (in the Basic_Model_Run) vignette

We note it is required by the journal to be able to reproduce all figures in the paper, however understandably the figures take too long to produce on a normal laptop since they perform extensive computations and parameter sweeps, so we have not reproduced them (with the exception of Figure 3, which was successfully reproduced). However, one minor comment is that in the script Shackleton26_simulations.R, line 31 needs to be updated (remove the _revised).

Paper

The paper is well written and introduces the software package well. We note some minor corrections:

- We recommend being explicit with what a ‘large population size’ means in line 192 (10 thousand? 100 thousand?), when comparing similarity between deterministic and stochastic outputs.

- The claim that mosquito biting rates increase with body surface area (line 145-6) would benefit from a citation. For example, this is the one commonly cited in OpenMalaria reference documentation when making the same claims: Port, G., Boreham, P., and Bryan, J. H. (1980). The Relationship of Host Size to Feeding by Mosquitoes of the Anopheles Gambiae Giles Complex (Diptera: Culicidae). Bulletin of Entomological Research 70, 133–144

- In the supplementary material S2.4, the authors say “We then allocate those individuals to each destination in proportion to their respective rates, ensuring that the total number leaving never exceeds the number present at the start of the time step” - however this does not seem to match the maths provided. Furthermore, this would be the wrong way to implement stochasticity, as instead the flow into each individual compartment should be a binomial realisation, not a proportion. The correct way does seem to be reflected in the maths and code provided, so we recommend updating the word description to also reflect this.

- Typo, line 121-2: “late instar lave → larvae”

- Line 170 is poorly worded, would benefit from being more specific: “accuracy compared to the individual-based malariasimulation model, but increases runtime” (or similar).

- Typo, line 221: “assumed to wane”

- Line 220: for completeness, it may be useful to provide the parameter for a mosquito that survives and successfully bites?

- SI 1: Figure S1.2 is floating - it has no reference in the text.

- SI 2: S2.2.4 typo: “clinincal recovery”

- SI 2: equation bottom of page 3 - we assume the integral should be with respect to time (i.e. add dt at the end).

*Nick Golding (Professor) and Yuval Berman (PhD student), University of Western Australia

**********

**Have the authors made all data and (if applicable) computational code underlying the findings in their manuscript fully available?**

The PLOS Data policy requires authors to make all data and code underlying the findings described in their manuscript fully available without restriction, with rare exception (please refer to the Data Availability Statement in the manuscript PDF file). The data and code should be provided as part of the manuscript or its supporting information, or deposited to a public repository. For example, in addition to summary statistics, the data points behind means, medians and variance measures should be available. If there are restrictions on publicly sharing data or code —e.g. participant privacy or use of data from a third party—those must be specified.

Reviewer #1: Yes

Reviewer #2: Yes

**********

PLOS authors have the option to publish the peer review history of their article (what does this mean?). If published, this will include your full peer review and any attached files.

If you choose “no”, your identity will remain anonymous but your review may still be made public.

**Do you want your identity to be public for this peer review?**  For information about this choice, including consent withdrawal, please see our Privacy Policy.

Reviewer #1: No

Reviewer #2: **Yes:**  Nick Golding and Yuval Bergman

[NOTE: If reviewer comments were submitted as an attachment file, they will be attached to this email and accessible via the submission site. Please log into your account, locate the manuscript record, and check for the action link "View Attachments". If this link does not appear, there are no attachment files.]

**Figure resubmission:**

**Reproducibility:**

To enhance the reproducibility of your results, we recommend that authors of applicable studies deposit laboratory protocols in protocols.io, where a protocol can be assigned its own identifier (DOI) such that it can be cited independently in the future. Additionally, PLOS ONE offers an option to publish peer-reviewed clinical study protocols. Read more information on sharing protocols at https://plos.org/protocols?utm_medium=editorial-email&utm_source=authorletters&utm_campaign=protocols

# Author comment: Author response to Decision Letter 2

**DOI:** 10.1371/journal.pcbi.1013687.r004

5 Aug 2026

Attachment Submitted filename: reviewer comments 2.docx

# Editor report: Decision Letter 2

**DOI:** 10.1371/journal.pcbi.1013687.r005

**Authors:** Roger Dimitri Kouyos, Oliver Eales

11 Aug 2026

Dear Dr Shackleton,

We are pleased to inform you that your manuscript 'malariasimple: An R package for fast simulations of malaria transmission' has been provisionally accepted for publication in PLOS Computational Biology.

Before your manuscript can be formally accepted you will need to complete some formatting changes, which you will receive in a follow up email. A member of our team will be in touch with a set of requests.

Please note that your manuscript will not be scheduled for publication until you have made the required changes, so a swift response is appreciated.

IMPORTANT: The editorial review process is now complete. PLOS will only permit corrections to spelling, formatting or significant scientific errors from this point onwards. Requests for major changes, or any which affect the scientific understanding of your work, will cause delays to the publication date of your manuscript.

Should you, your institution's press office or the journal office choose to press release your paper, you will automatically be opted out of early publication. We ask that you notify us now if you or your institution is planning to press release the article. All press must be co-ordinated with PLOS.

Thank you again for supporting Open Access publishing; we are looking forward to publishing your work in PLOS Computational Biology.

Best regards,

Oliver Eales

Academic Editor

PLOS Computational Biology

Roger Kouyos

Section Editor

PLOS Computational Biology

***********************************************************

# Editor report: Acceptance letter

**DOI:** 10.1371/journal.pcbi.1013687.r006

**Authors:** Roger Dimitri Kouyos, Oliver Eales

PCOMPBIOL-D-25-02244R2

malariasimple: An R package for fast simulations of malaria transmission

Dear Dr Shackleton,

I am pleased to inform you that your manuscript has been formally accepted for publication in PLOS Computational Biology. Your manuscript is now with our production department and you will be notified of the publication date in due course.

The corresponding author will soon be receiving a typeset proof for review, to ensure errors have not been introduced during production. Please review the PDF proof of your manuscript carefully, as this is the last chance to correct any errors. Please note that major changes, or those which affect the scientific understanding of the work, will likely cause delays to the publication date of your manuscript.

Soon after your final files are uploaded, unless you have opted out, the early version of your manuscript will be published online. The date of the early version will be your article's publication date. The final article will be published to the same URL, and all versions of the paper will be accessible to readers.

For Research, Software, and Methods articles, you will receive an invoice from PLOS for your publication fee after your manuscript has reached the completed accept phase. If you receive an email requesting payment before acceptance or for any other service, this may be a phishing scheme. Learn how to identify phishing emails and protect your accounts at https://explore.plos.org/phishing.

Thank you again for supporting PLOS Computational Biology and open-access publishing. We are looking forward to publishing your work!

With kind regards,

Janani Seenivasan

PLOS Computational Biology | Carlyle House, Carlyle Road, Cambridge CB4 3DN | United Kingdom ploscompbiol@plos.org | Phone +44 (0) 1223-442824 | ploscompbiol.org | @PLOSCompBiol
